# Deletion of Specific Sphingolipids in Distinct Neurons Improves Spatial Memory in a Mouse Model of Alzheimer’s Disease

**DOI:** 10.3389/fnmol.2018.00206

**Published:** 2018-06-20

**Authors:** Silke Herzer, Cassidy Hagan, Johanna von Gerichten, Vanessa Dieterle, Bogdan Munteanu, Roger Sandhoff, Carsten Hopf, Viola Nordström

**Affiliations:** ^1^Division of Cellular and Molecular Pathology, German Cancer Research Center, Heidelberg, Germany; ^2^Interdisciplinary Center for Neurosciences, Heidelberg University, Heidelberg, Germany; ^3^Department of Microbiology, Immunology, and Pathology, Colorado State University, Fort Collins, CO, United States; ^4^Lipid Pathobiochemistry Group, German Cancer Research Center, Heidelberg, Germany; ^5^Center for Mass Spectrometry (CeMOS), University of Heidelberg and Mannheim University of Applied Sciences, Mannheim, Germany

**Keywords:** Alzheimer’s disease, gangliosides, spatial memory, spine density, glial cells

## Abstract

Alzheimer’s disease (AD) is characterized by progressive neurodegeneration and a concomitant loss of synapses and cognitive abilities. Recently, we have proposed that an alteration of neuronal membrane lipid microdomains increases neuronal resistance toward amyloid-β stress in cultured neurons and protects from neurodegeneration in a mouse model of AD. Lipid microdomains are highly enriched in a specific subclass of glycosphingolipids, termed gangliosides. The enzyme glucosylceramide synthase (GCS) catalyzes the rate-limiting step in the biosynthesis of these gangliosides. The present work now demonstrates that genetic GCS deletion in subsets of adult forebrain neurons significantly improves the spatial memory and counteracts the loss of dendritic spines in the hippocampal dentate gyrus of 5x familial AD mice (5xFAD//*Ugcg*f/f//Thy1-CreERT2//EYFP mice), when compared to 5xFAD//*Ugcg*f/f littermates (5xFAD mice). Aberrantly activated glial cells and their expression of pro-inflammatory cytokines have emerged as the major culprits for synaptic loss in AD. Typically, astrocytic activation is accompanied by a thickening of astrocytic processes, which impairs astrocytic support for neuronal synapses. In contrast to 5xFAD mice, 5xFAD//*Ugcg*f/f//Thy1-CreERT2//EYFP display a less pronounced thickening of astrocytic processes and a lower expression of tumor necrosis factor-α and interleukin 1-α in the hippocampus. Thus, this work further emphasizes that GCS inhibition may constitute a potential therapeutic target against AD.

## Introduction

Alzheimer’s disease (AD) leads to progressive loss of cognitive abilities. Histopathologically, affected brain regions typically display extracellular amyloid-β (Aβ) plaques, intracellular neurofibrillary tangles (Tanzi, 2013), and synapse loss (Hamos et al., 1989). For a long time, the “amyloid cascade hypothesis” proposed that Aβ plaque deposition is the main causative component of AD (Hardy and Higgins, 1992). However, the lack of progress in developing novel disease-modifying therapies based on Aβ plaque reduction (Head et al., 2008) led to reformulation and refinements of this hypothesis. While aberrant expression and processing of amyloid precursor protein (APP) are causative for certain cases of human familial AD, empiric data do not support a causative role for late-onset AD cases (Morris et al., 2014). Novel hypotheses have rather suggested that more complex processes such as neuroinflammation (Krstic and Knuesel, 2013) as well as the build-up of neurotoxic Aβ oligomers (Lambert et al., 1998) are mainly involved in AD onset and progression.

Synaptic loss is an early hallmark of AD (Hamos et al., 1989; Eroglu and Barres, 2010). In a healthy brain, synapses are intimately associated with glial cells, which provide trophic support and modulate synaptic function (Eroglu and Barres, 2010). Synapse loss and the concomitant cognitive decline in AD go along with progressing neuroinflammation, which is characterized by abnormal activation of astrocytes and microglia (Chung et al., 2015). Aberrantly activated glial cells highly express inflammatory mediators and contribute to the cognitive decline in AD (Morales et al., 2014), in part due to increased oxidative stress (Hensley, 2010), and in part due to the fact that hyperreactive glial cells no longer adequately provide trophic support and homeostatic maintenance functions for the neurons (Oberheim et al., 2008; Krstic and Knuesel, 2013). Specifically, thickening of astrocytic processes is supposed to cause a retraction of the astrocytic end-feet from synapses, thereby destabilizing the synaptic integrity (Barrow and Small, 2007). Moreover, activated “harmful” astrocytes are suggested to counteract the formation of new synapses and synapse maintenance (Liddelow et al., 2017) and thereby contribute to Aβ-induced synapse loss. Synapse loss is reflected by a decrease in spine density (Zhang et al., 2014). It has to be noted, however, that neuroinflammation *per se* may not be regarded as solely harmful (Hensley, 2010). For example, subsets of activated glial cells phagocytose Aβ aggregates and thereby contribute to Aβ clearance (Boche and Nicoll, 2008; Hensley, 2010). Moreover, Liddelow et al. (2017) recently described that, in contrast to “harmful” reactive astrocytes (so-called A1 astrocytes), also “neuroprotective” reactive astrocytes (so-called A2 astrocytes) are induced mainly by ischemic lesions. A2 astrocytes display a different gene expression pattern than A1 astrocytes and produce factors that promote survival and growth of neurons.

Many cellular processes such as APP processing, synaptic transmission, and transmembrane receptor signaling take place at the neuronal plasma membrane. Plasma cell membranes are built up by various lipid species, such as phospholipids, sterols, and sphingolipids. However, these lipids are not evenly distributed within the membranes. Dynamic lipid microdomains in neuronal membranes are highly enriched in glucosylceramide synthase (GCS; gene: *Ugcg*)-derived gangliosides, a sub-class of glycosphingolipids. These gangliosides can directly modulate the activity of transmembrane receptors (Nordström et al., 2013; Herzer et al., 2015, 2016) and other membrane processes such as endocytosis (Ewers et al., 2010). Ganglioside biosynthesis is altered in AD brains (Ariga et al., 2008). It has been shown that gangliosides GM1 and GM2 accumulate in frontal and temporal cortex of AD patients (Pernber et al., 2012), while more complex gangliosides GQ1bα and GT1aα accumulate in brains of transgenic AD mouse models (Ariga et al., 2010). Moreover, gangliosides are suggested to destabilize mature Aβ fibrils and resolubilize them to synaptotoxic protofibrils (Martins et al., 2008) and act as seeds for Aβ fibril formation (Yanagisawa et al., 1995). We have shown that pharmacological GCS inhibition and subsequent reduction of gangliosides increase the resistance of cultured neurons toward oligomeric Aβ stress and protect from Aβ-induced loss of neuronal insulin receptors (IRs) (Herzer et al., 2016; Nordström and Herzer, 2017). Moreover, we have found less neurodegeneration in transgenic 5x familial AD (5xFAD) mice (Oakley et al., 2006) with a tamoxifen-inducible GCS deletion in forebrain neurons (Herzer et al., 2016). Thus, we hypothesized that GCS might be a potential novel therapeutic target for the treatment of AD.

The present study addresses the question of whether inhibition of ganglioside biosynthesis can also improve the cognitive capacity and counteract spine loss in 5xFAD mice. Intriguingly and in line with our previous work (Herzer et al., 2016), behavioral tests indicate that spatial memory is improved in 5xFAD mice which harbor inducible GCS deletion in subsets of adult forebrain neurons. Additionally, these mice are protected from Aβ-induced spine loss. Thus, the current work emphasizes that GCS inhibition and subsequent ganglioside reduction may constitute promising novel cellular targets against AD.

## Materials and Methods

### Mice

*Ugcg*f/f//Thy-1-CreERT2 mice were crossed to 5xFAD mice (The Jackson Laboratory) to generate 5xFAD-Thy1-Cre, 5xFAD, Thy1-Cre- and control littermates. All mice were backcrossed to C57BL6 (>12 generations). Tamoxifen injections occurred 9 weeks after birth as previously described (Nordström et al., 2013). All mouse groups analyzed (controls, 5xFAD, Thy1-Cre, 5xFAD-Thy1-Cre) received tamoxifen injections. Mice received solvent injections without tamoxifen when indicated. This study was carried out in accordance with the recommendations of the DKFZ internal committees and national regulations for animal experiments. The protocol was approved by the Regierungspräsidium Karlsruhe (Germany).

### *In Situ* Hybridization

5 μM sagittal brain sections were prepared under RNase-free conditions. *In situ* hybridization (ISH) was performed by using a commercially available kit (RNAscope 2.0 HD Brown, Advanced Cell Diagnostics (ACD)) according to the manufacturer’s guidelines and as described earlier by Herzer et al. (2016). Slides were exposed to either a probe recognizing *Ugcg* (ISH probe targeting region 653–1108 of mouse *Ugcg* mRNA (ACD)) or a negative control probe DAPB (ACD). For detection of targeted neurons, sections were stained with rabbit-α-GFP (1:25, Abcam) that also detects YFP. The secondary antibody was donkey-α-rabbit Alexa-Fluor488 (1:100, Invitrogen). For additional detection of glial cells, sections were additionally stained with mouse-α-GFAP (1:200, Millipore) and secondary goat-α-mouse Alexa-Fluor555 (1:200, Invitrogen). Slides were analyzed with a bright field/fluorescence microscope (Keyence).

For improved visibility, brown ISH spots were digitally converted to red fluorescence by adjusting the brightfield channel picture as follows: the picture was inverted and the green and blue channels were adjusted to “zero” output in order to visualize the brown ISH spots as red fluorescent spots. For comparison, the original channel overlay pictures showing brown spots have been included in the respective indicated Supplementary Figures. In case of *Ugcg* mRNA detection in glial cells (brown spots were converted to magenta by adjusting the brightfield image as follows: the picture was inverted, converted to 8 bit, and magenta was chosen in the LUT settings. For comparison, the original channel overlay pictures showing brown spots have been included in the indicated Supplementary Figure.

### Active Place Avoidance Test

Female mice (control, 5xFAD, Cre, and 5xFAD-Thy1-Cre littermates) were used for cognitive tests at an age of 9 months. The active place avoidance test was performed as described earlier (Krieger et al., 2012). Mice were placed onto a rotating circular platform (*r* = 40 cm, 1 rpm), which was surrounded by a transparent wall. Stationary cues visible for the mice outlined a defined zone (R0 shock area). Upon entering the R0 area, mice received a 0.4-mA shock, followed by further identical shocks every 1.5 s when mice stayed in the R0 area. As the platform rotated, it was not possible for mice to avoid the R0 sector due to a passive strategy. After a 10-min habituation phase (trial 1) without shocks, eight 10 min trials (trials 2–9) were conducted with pauses of 30 min, where animals were placed back into their cages (training phase). Mice were continuously tracked throughout the trials by a video camera and the analysis was performed automatically by the SYGNIS tracker software. The learning performance was monitored and displayed as R0 visits/distance run, latency to first R0 zone entry, time spent in R0, and number of shocks received during each trial.

After 24 h, one recall trial without electrical shocking was carried out to analyze memory retention. The above-mentioned parameters were analyzed by the SYGNIS tracker software.

### Spontaneous Y-Maze Alternation

The spontaneous Y-maze alternation test was performed as described earlier (Oakley et al., 2006). Nine-month-old mice were placed into the center of a symmetrical Y-maze apparatus. Their behavior was recorded for 5 min. The sequence of arm entries (A, B, or C) was monitored and the % spontaneous Y-maze alternation was calculated as follows (Oakley et al., 2006): number of triads with sequential entries into all three arms/maximum possible alternations (total arm entries - 2) × 100.

### Brain Sections

Paraffin and cryosections were prepared according to standard procedures, as described earlier by Nordström et al. (2013) and Herzer et al. (2015, 2016). For paraffin sections, brain hemispheres of mice (transcardially perfused with 4% PFA) were incubated in 4% PFA (4°C, 7 days) and subsequently embedded in paraffin according to standard procedures. 5 μM paraffin sections were subsequently prepared for further stainings. For cryosections, brain hemispheres of mice (transcardially perfused with 4% PFA) were immersion-fixed in 4% PFA (4°C, 1 day) and subsequently subjected to cryoprotection by immersion in 30% sucrose. 40 μM free-floating cryosections were prepared for further stainings.

### Immunofluorescence and Histochemistry

Immunostainings of brain sections and image acquisition were performed according to standard procedures as described earlier by Nordström et al. (2013) and Herzer et al. (2015, 2016). Blocking (RT, 1 h) and antibody incubations (4°C, o/n) occurred in 1%BSA/0.05% Triton-X/PBS. Primary antibodies were mouse-α-Aβ (6E10; 1:200, Covance), rabbit-α-Aβ_42_ (1:50, Millipore), rabbit-α-GFP (1:50, Invitrogen), rabbit-α-Iba1 (1:200, WAKO), mouse-α-GFAP (1:200, Millipore), and oligomer-specific antibody rabbit-α-A11 (1:50, Invitrogen). Secondary antibodies used were donkey-α-mouse Alexa-Fluor488, donkey-α-rabbit Alexa-Fluor488, goat-α-rabbit Alexa-Fluor555, goat-α-mouse Alexa-Fluor555 (1:200, Invitrogen). For immunohistochemistry, subsequent staining was performed with biotin-α-mouse or biotin-α-rabbit (1:150, Jackson Immuno Research) and horseradish peroxidase streptavidin (1:200, Vector). For histochemistry, AEC reagent (BioGenex) was used. Coverslips were mounted with ProLongGold^®^ (Invitrogen) and subsequently analyzed by fluorescence or brightfield (Keyence) or confocal microscopy (Leica).

### Analysis of Spine Density and Image Processing

The following mouse groups (> 10 months of age) were analyzed: non-induced *Ugcg*f/f//Thy1-Cre-ERT2//EYFP (n.i. control; no Cre activity but EYFP expression), non-induced 5xFAD//*Ugcg*f/f//Thy1-Cre-ERT2//EYFP (n.i. 5xFAD; no Cre activity but EYFP expression), tamoxifen-induced *Ugcg*f/f//Thy1-Cre-ERT2//EYFP (Cre; Cre activity and EYFP expression), and tamoxifen-induced 5xFAD//*Ugcg*f/f//Thy1-Cre-ERT2//EYFP mice (5xFAD-Thy1-Cre; Cre activity and EYFP expression). Non-induced mice received solvent injections without tamoxifen instead. Paraffin sections were prepared and spine density was analyzed as described earlier (Zhang et al., 2014) with minor modifications. After transcardial perfusion (4% PFA), brains were harvested and 5 μm paraffin sections were prepared. Prior to immunofluorescence, antigen retrieval was performed in a pressure heater (120°C, 25 min) in citrate buffer (0.1M C_6_H_9_Na_3_O_9_/0.1M C_6_H_10_O_8_, pH 6.0). Immunofluorescence was performed as described above, by using rabbit-α-GFP (1:50, Invitrogen) and secondary donkey-α-rabbit Alexa-Fluor488 (1:200, Invitrogen). Z-stack images were acquired with a confocal microscope (Leica, TCS SP5II). The spine morphology was reconstructed from serial z-stack images by ImageJ (NIH), by background subtraction (rolling ball radius: 10 pixels) followed by despeckle and subsequent Z-stack projection. The spine density was analyzed as described earlier (Zhang et al., 2014), by counting spines that emerged perpendicular to the dendritic shaft. The number of spines was counted in relation to the dendrite length (dendritic ROI). Image analysis occurred blind toward the genotypes. In case of n.i. 5xFAD and 5xFAD-Thy1-Cre mice, spines were analyzed in neuronal processes that were located adjacent to plaques (approximated distance between 20 and 30 μm).

### Morphometric Analysis of Amyloid Plaques, A11 Content, Intracellular 6E10, and Astrocytic Process Width

Paraffin sections from mouse brains (9 months of age) were prepared and subjected to immunostaining as described above. The location of sections was determined with the help of the Paxinos Brain Atlas. The number of sections and individual mice is indicated in the respective figure legends. Morphometric analyses of plaques and A11 were carried out with ImageJ (NIH).

For Aβ_42_ plaque morphology, figures were equally adjusted in threshold (ImageJ). For 6E10/A11 morphometry, solely A11 signal present in 6E10-positive plaques was included in the analysis. GFAP-stained astrocytes in the hippocampal dentate gyrus (DG) were analyzed by z-stack fluorescence microscopy (Keyence). In astrocytes, the width of all astrocytic processes emerging from the soma was measured as described earlier (Sun et al., 2010). The width of each astrocytic process was analyzed at its respective emergence point from the soma by a morphometric measurement (ImageJ, NIH). The investigator was blinded toward the genotypes.

### Quantitative mRNA Analysis

Quantitative mRNA expression analysis was performed as described previously by Nordström et al. (2013). RNA was extracted from tissue samples with the Ambion^®^PARIS^TM^ kit (Thermo Fisher Scientific) according to the manufacturer’s guidelines. RNA was reverse transcribed by Superscript II Reverse Transcriptase (Invitrogen) and cDNA was quantified using the LC FastStart DNA Master SYBR Green I kit (Roche) according to the manufacturer’s guidelines and the Light Cycler (Roche). Expression levels were normalized to the expression of the housekeeping gene glyceraldehyde-3-phosphate dehydrogenase (GAPDH). The following primers were used: IL1-α forward: 5′-CCTACTCGTCGGGAGGAGAC-3′; IL1-α reverse: 5′-GGTGGCAATAAACAGCTCTGG-3′; TNF-α forward: 5′-AAGCATGATCCGCGACGTGGA-3′; TNF-α reverse: 5′-TTGCTACGACGTGGGCTACA-3′ IL1-β forward: 5′-TGCAGTGGTTCGAGGCCTAA-3′; IL1-β reverse: 5′-GCTGCTGCGAGATTTGAAGCTG-3′; GAPDH forward 5′-ACTCCCACTCTTCCACCTTC-3′; GAPDH reverse: 5′-GGTCCAGGGTTTCTTACTCC-3′.

### X-Gal Stainings

*Ugcg*f/+//Thy1CreERT2/EYFP mice were crossbred to R26R Rosa LacZ reporter mice to generate Rosa26//*Ugcg*f/f//Thy1CreERT2/EYFP (Rosa26//Thy1-Cre) mice as described earlier (Nordström et al., 2013). Three days after induction, animals were sacrificed, brains were removed and frozen on dry ice. X-Gal staining was performed as described previously (Nordström et al., 2013).

### Liquid Chromatography-Coupled Tandem Mass Spectrometry of Ganglioside Species

Lipids were extracted from brain tissue and glycosphingolipids were purified and separated into neutral and acidic glycosphingolipids as described previously by Nordström et al. (2013). Aliquots corresponding to 2.5 mg protein/ml were mixed with internal lipid standard for analysis by LC-MS/MS using an Aquity I-class UPLC and a Xevo TQ-S “triple-quadrupole” MS instrument, both from Waters. Using a CORTECS HILIC column (2.1 mm × 100 mm; 1.7 μm, Waters), gangliosides were measured in positive mode with a gradient between 100% solvent A (90% acetonitrile) and 100% solvent B (50% acetonitrile), both containing 10 mM ammonium formate as additive. Gangliosides were analyzed by multireaction monitoring (MRM) with MS/MS-transitions for both, the protonated molecular ion and the corresponding ammonia adduct at optimized collision energy (Supplementary Figure 1A). Transitions reflect the majority of ganglioside species, which contain a d18:1 long chain base (C18-sphingosine) and C16 to C24 fatty acyl chain length. GM3 (d18:1; C19:0) was used as internal standard and purified human gangliosides as external standard (Supplementary Figure 1B).

### MALDI-Imaging of Brain Slices

Frozen mouse brains from 8-month-old mice were processed and MALDI matrix application was applied as previously described (Munteanu et al., 2014) with minor adjustments. Briefly, 60 mg/mL _s_DHB in ACN/ddH_2_O/TFA (40/60/0.5 v/v/v) was applied to tissue sections by spray coating using the following parameters of the SunCollect device (SunChrom, Friedrichsdorf, Germany): two initial matrix layers using a flow-rate of 10 μL/min followed by three layers of 15 μL/min at 150 mm/min were chosen to ensure a homogeneous matrix layer on tissue slides. A drying step of 3 min was introduced between each cycling step. MALDI-MSI measurements were performed using the Rapiflex MALDI-TOF MS (Bruker Daltonics) in positive linear mode in the mass to charge range of m/z 2000-20,000 using the FlexImaging 5.0 (Bruker Daltonics). Prior to analysis, the acquisition method was calibrated using the protein calibration standard I (Bruker Daltonics). A total of 250 laser shots were accumulated per raster spot at a laser width of 20 × 20 μm at a lateral resolution of 20 μm and 10 kHz laser acquisition speed. In addition, baseline subtraction was performed and images were visualized after total ion count normalization in FlexImaging software. In order to evaluate potential shifts in Aβ ratios (40 vs. 42) covering distinct brain regions, regions of interest (ROIs) for each hippocampal lobe were manually marked in SCiLS software (Bruker Daltonics) according to H&E staining of follow-up sections. Average spectra for marked ROIs were further processed by baseline subtraction, smoothing and peak picking using default predefined parameters. Intensities corresponding for Aβ 1–40 and 1–42 within a mass window of ±5 Da were exported and the ratio (40 vs. 42) calculated in Excel (Microsoft). For statistical evaluation and visualization, ratios were transferred to Prism 5.0 software (Graphpad Software).

### Statistics

Data are presented as mean ± SEM. Statistical analysis was done with Graph Pad Prism and specified in respective figure legends. Comparison of mean values from two groups was performed by an unpaired two-tailed Student’s t-test. Results were marked with (^∗^) if *P* ≤ 0.05, (^∗∗^) if *P* ≤ 0.01, (^∗∗∗^) if *P* ≤ 0.001, or (#) if *P* ≤ 0.1. Otherwise, mean values of more than two groups were analyzed by one-way ANOVA with Tukey’s test for multiple comparisons (95% confidence interval), as indicated in the respective figure legends.

## Results

### Generation of 5xFAD Mice With Inducible GCS Deletion in Distinct Neuronal Subsets

**FIGURE 1:**
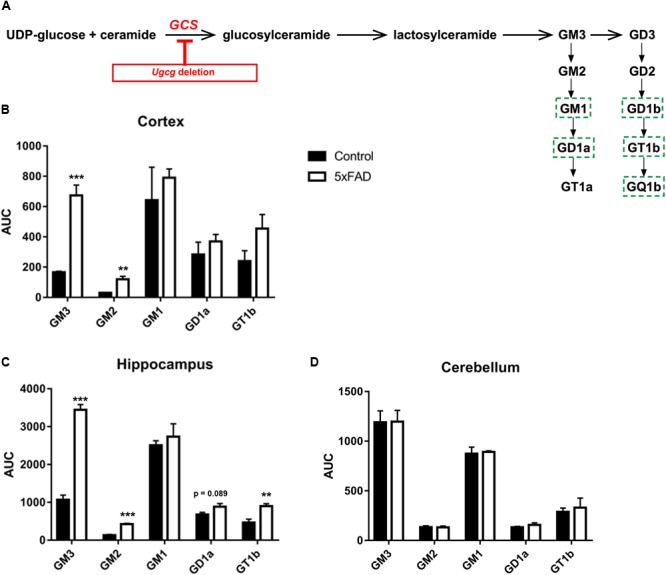
GCS deletion in distinct subsets of adult neurons in a 5xFAD mouse model. **(A)** Schematic representation of the biosynthesis of the ganglioside a- and b-series. Glucosylceramide synthase (GCS, gene: *Ugcg*) is the key enzyme in ganglioside biosynthesis. Major neuronal gangliosides are outlined. Genetic *Ugcg* deletion inhibits *de novo* ganglioside biosynthesis. **(B,C)** Mass spectrometry analysis of ganglioside levels shows that gangliosides GM3, GD3, and GT1b are increased in cerebral cortex **(B)** and hippocampus **(C)** of 5xFAD mice. AUC: area under curve, corresponding to the peak area normalized to the internal standard. **(D)** Ganglioside levels are unchanged in the cerebellum of 5xFAD mice, which is not affected by Aβ pathology (*n* = 4 (cortex and hippocampus) or 3 (cerebellum) 14-month-old mice per group). Statistical analysis was performed by unpaired two-tailed Student’s t-test. Means ± SEM. (^∗∗^) if *P* ≤ 0.01, or (^∗∗∗^) if *P* ≤ 0.001.

GCS is the key enzyme in ganglioside biosynthesis (**Figure 1A**). Earlier reports have shown that ganglioside metabolism is altered in brains of AD mouse models (Ariga et al., 2008, 2010, 2013). Moreover, deletion of GD3 synthase was reported to exert beneficial effects on the cognition of APP/PSEN1 transgenic mice (Bernardo et al., 2009), and GCS deletion in adult forebrain neurons counteracts neurodegeneration (Herzer et al., 2016). Indeed, mass spectrometry shows that the levels of ganglioside species GM3, GM2, and GT1b are increased in cortex and hippocampal tissue of 14-month-old 5xFAD mice (**Figures 1B,C**). Moreover, GD1a also shows a tendency to be elevated in 5xFAD mice (**Figures 1B,C**). On the other hand, ganglioside levels in the cerebellum, which is not affected by Aβ pathology in 5xFAD mice, are normal (**Figure 1D**).

**FIGURE 2:**
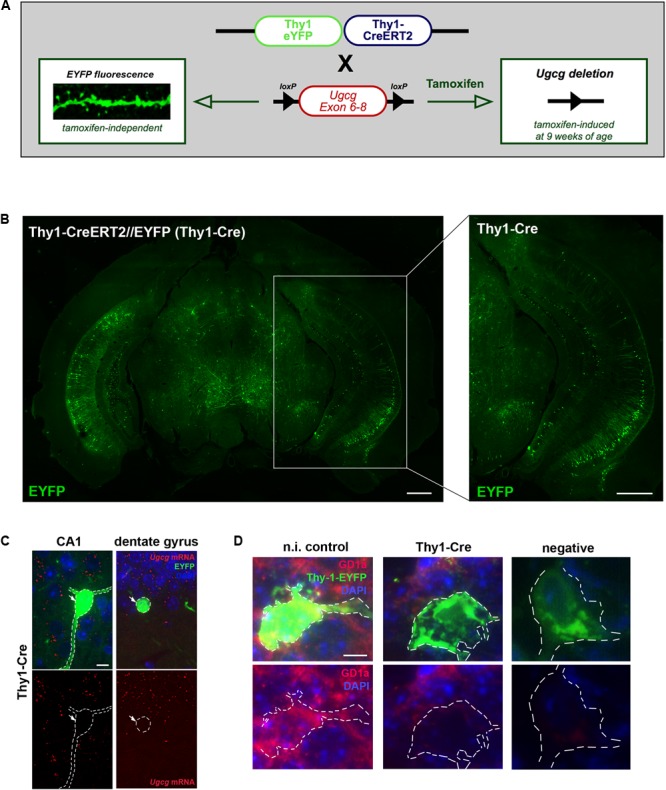
Generation of tamoxifen-inducible Thy1-Cre mice with GCS deletion in subsets of adult forebrain neurons. **(A)** The targeting construct in Thy1-CreERT2/EYFP mice comprises one Thy1 copy that drives tamoxifen-inducible Cre recombinase and the second Thy1 copy that drives tamoxifen-independent EYFP in targeted neurons. **(B)** EYFP fluorescence shows that neurons in the hippocampal regions CA1, CA2, and dentate gyrus are targeted by the construct (bregma –3.08 mm (coronal), scale bars = 200 μm). **(C)** An *in situ* hybridization (ISH) of tamoxifen-induced *Ugcg*f/f//Thy1-CreERT2/EYFP (Thy1-Cre) mice confirms that Cre-targeted neurons are devoid of GCS expression. The original brown ISH dots have been converted to red fluorescence, as described in Materials and Methods section (scale bar = 10 μm). For comparison, the original images are depicted in Supplementary Figure 2C. **(D)** Immunofluorescence shows that ganglioside GD1a is absent in Cre-targeted and fluorescent neurons of tamoxifen-induced Thy1-Cre mice. On the contrary, non-induced Thy1-Cre mice (n.i. control) mice only receiving solvent injections without tamoxifen display GD1a expression in EYFP-fluorescent neurons, because Cre activity is not induced in this case (scale bar = 5 μm).

To generate mice with inducible GCS deletion in adult neurons, *Ugcg*f/f mice were crossbred with mice expressing tamoxifen-inducible Cre recombinase and tamoxifen-independent EYFP fluorescence under the neuron-specific *Thy-1* promoter in subsets of forebrain neurons (Young et al., 2008). In the targeting construct, one *Thy-1* promoter copy drives tamoxifen-inducible Cre activity, while the second one drives tamoxifen-independent EYFP expression (Young et al., 2008) (**Figure 2A**). *Ugcg*f/f//Thy1-CreERT2/EYFP mice (Thy1-Cre mice) (Jennemann et al., 2005; Young et al., 2008) were crossbred with 5xFAD mice (Oakley et al., 2006) to generate 5xFAD-*Ugcg*f/f//Thy1-CreERT2/EYFP mice (5xFAD-Thy1-Cre mice). In Thy1-Cre mice, Cre activity is, amongst others, present in hippocampal neurons (**Figure 2B**), which is important since the hippocampus is highly affected by AD pathology. Thy1-Cre mice display diverse Cre targeting in various neuronal subsets (Young et al., 2008). Sparse targeting is present in the cerebral cortex, thalamus, striatum, and brain stem, whereas moderate targeting is observed in hippocampus, amygdala, and in p1 reticular formation (Young et al., 2008; **Figure 2B**). Since the dorsal part of the hippocampus is important for cognitive function (O’Keefe and Nadel, 1978), we estimated the rate of Cre-positive neurons in sagittal sections of the dorsal hippocampus (sagittal sections located between lateral 0.96 and 1.2 mm) by counting the number of NeuN/EYFP-double-positive neurons. We observed that targeting is diverse, and found that at least ∼17.3% of neurons in the DG are Cre-positive, whereas for example the CA1 region in this region displays a rather low targeting rate of only ∼1.97% Cre-positive neurons (Supplementary Figure 2A). The functionality of the Cre recombinase in targeted neurons has been confirmed by tamoxifen-induced Rosa26//Thy1-Cre reporter mice (Supplementary Figure 2B). Moreover, ISH shows that *Ugcg* is stably deleted in targeted neurons (**Figure 2C** and Supplementary Figures 2C,D), and the consequent inhibition of ganglioside biosynthesis is visualized by reduced GD1a immunofluorescence (**Figure 2D**).

We have furthermore monitored lipid levels in hippocampal tissue of all mouse groups by liquid chromatography-coupled tandem mass spectrometry (Supplementary Figure 2E). Sphingomyelin was slightly, but not significantly reduced in 5xFAD mice and it was unaltered in the other mouse groups. Ceramide and lactosylceramide levels were also slightly, but not significantly elevated in 5xFAD mice, but also in 5xFAD-Thy1-Cre mice. Hexosylceramides, including galactosylceramide and glucosylceramide, were slightly lower in Thy1-Cre and 5xFAD-Thy1-Cre mice. Importantly and unlike *Ugcg*f/f//CamK-CreERT2 mice described in our previous studies (Nordström et al., 2013; Herzer et al., 2015), 9-month-old Thy1-Cre mice do not develop obesity (Supplementary Figure 2F).

Nine-month-old 5xFAD mice display Aβ plaques in, amongst others, the cerebral cortex, the hippocampal DG, and hippocampal CA1. An initial morphometric analysis of sections stained with 6E10 (Supplementary Figure 3A) shows that the mean 6E10-positive Aβ plaque size (Supplementary Figure 3B) and number (Supplementary Figure 3C), as well as the 6E10-positive amyloid plaque load (Supplementary Figure 3D) in sagittal hippocampal sections between lateral 0.96 and 1.2 mm of 5xFAD mice do not differ from 5xFAD-Thy1-Cre mice.

**FIGURE 3:**
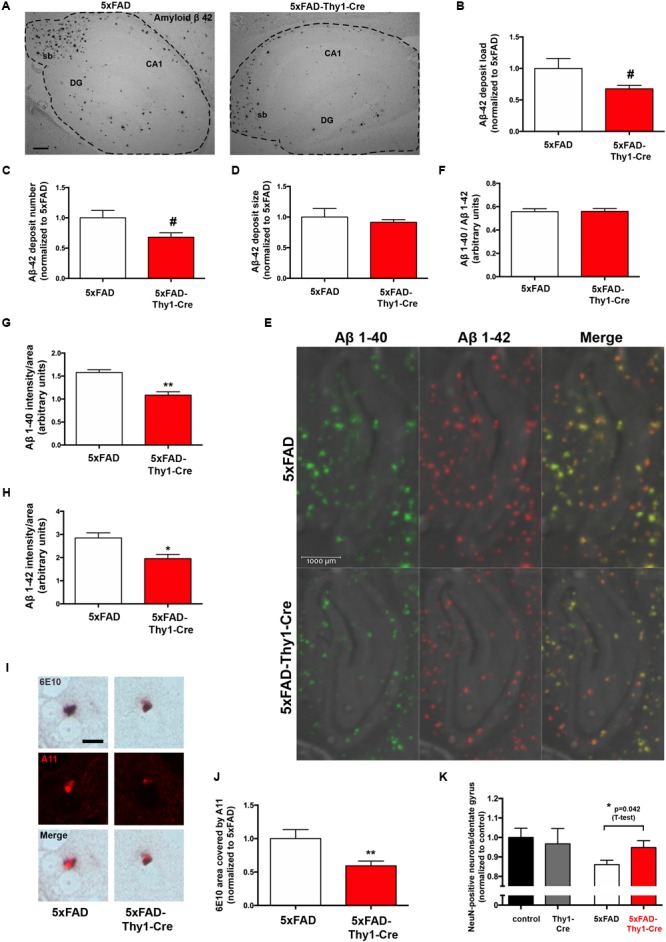
Lower Aβ_42_ deposition and occurrence of oligomeric Aβ in 6E10-positive plaques of 5xFAD-Thy1-Cre mice. **(A)** Hippocampal sections (between lateral 0.96 and 1.2 mm (sagittal)) were stained with an antibody detecting specifically Aβ_42_ (sb = subiculum, DG = dentate gyrus, CA1 region; scale bar = 300 μm). Morphometry of these sections shows that **(B)** the Aβ_42_ load and **(C)** the Aβ_42_ deposit number are decreased in 5xFAD-Thy1-Cre mice. **(D)** The size of Aβ_42_ deposits does not differ between 5xFAD and 5xFAD-Thy1-Cre mice (*n* = 4 5xFAD or 5 5xFAD-Thy1-Cre brain sections derived from 4 to 5 mice, respectively; 1251 (5xFAD) and 1064 (5xFAD-Thy1-Cre) Aβ_42_ deposits were analyzed in total). **(E)** MALDI imaging has been performed on hippocampal sections located between bregma –1.82 and –1.94 mm (coronal) in order to distinguish Aβ_40_ and Aβ_42_ signals. **(F)** The Aβ_40_/Aβ_42_ ratio in total hippocampal sections is not altered, as determined by MALDI imaging. However, both Aβ_40_ **(G)** and Aβ_42_ **(H)** signal intensities normalized to the area are lower in 5xFAD-Thy1-Cre mice (*n* = 4 (5xFAD) and 6 (5xFAD-Thy1-Cre) hippocampal hemispheres from two 5xFAD and three 5xFAD-Thy1-Cre mice; statistical analysis was performed by unpaired two-tailed Student’s t-test). **(I)** A double staining depicts plaques located in striatum (lateral 0.96–1.2 mm (sagittal)) which are positive for both 6E10 and the oligomer-specific antibody A11, indicating oligomeric Aβ species located within Aβ plaques (scale bar = 10 μm). **(J)** The 6E10 plaque area that is covered by A11 signal is decreased in 5xFAD-Thy1-Cre mice (n = 76 (5xFAD) and 83 (5xFAD-Thy1-Cre) 6E10-positive plaques in sections derived from five mice each). **(K)** NeuN stainings were performed on hippocampal sections located between lateral 0.96 and 1.2 mm (sagittal). The numbers of NeuN-positive neurons (normalized to control) are depicted (*n* = 7 control, 8 Thy1-Cre, 10 5xFAD, and 7 5xFAD-Thy1-Cre sections derived from four control or five Thy1-Cre, 5xFAD, 5xFAD-Thy1-Cre mice). Statistical analysis was performed by one-way ANOVA with Tukey’s test for multiple comparison (confidence interval = 95%). An additional statistical comparison of the means of the two primary groups of interest (5xFAD vs. 5xFAD-Thy1-Cre) was performed by a two-tailed Student’s *t*-test and the respective *p*-value is depicted in the figure. Statistics for **(B–D,E,G,H,J)** were performed by unpaired two-tailed Student’s t-test. Means ± SEM. (#) if *P* ≤ 0.1, (^∗^) if *P* ≤ 0.05, or (^∗∗^) if *P* ≤ 0.01.

However, it has to be noted that the 6E10 antibody tends to have a certain cross-reactivity with APP and APP cleavage products apart from Aβ (Hunter and Brayne, 2017), which may have blunted a potential difference. Therefore, we have performed additional morphometry with sections stained with an antibody specifically recognizing Aβ_42_ (**Figure 3A**). Interestingly, in this approach we detect a slight decrease in the Aβ_42_ load (**Figure 3B**) and the number of Aβ_42_ deposits (**Figure 3C**), whereas the size of Aβ_42_ deposits is unchanged (**Figure 3D**). Furthermore, an analysis by MALDI-imaging was performed to distinguish Aβ_42_ and Aβ_40_ species. This revealed no significant difference in the Aβ_40_/Aβ_42_ ratio in hippocampal sections located between bregma -1.82 and -1.94 mm (coronal) of 5xFAD and 5xFAD-Thy1-Cre mice (**Figures 3E,F** and Supplementary Figure 4A). Importantly, the MALDI analysis confirmed that the signal intensity is slightly decreased for Aβ_42_, but also for Aβ_40_ in the hippocampus of 5xFAD-Thy1-Cre mice (**Figures 3G,H**). On the other hand, an ELISA show that total Aβ_1-42_ and Aβ_1-40_ contents are not altered in cerebral cortex of 5xFAD-Thy1-Cre mice (Supplementary Figures 4B,C), a region that is, with few exceptions, not targeted by Cre activity (**Figure 2B**). Apart from the deposition in Aβ plaques, the occurrence of intracellular Aβ has been reported in AD mouse models (Bittner et al., 2010). At the age of 9 months, we observe the occurrence of intracellular 6E10 signal in our 5xFAD mouse model mainly in the hippocampal CA1 region. Intracellular 6E10 signals in this region are indistinguishable between 5xFAD and 5xFAD-Thy1-Cre mice (Supplementary Figures 4D,E). However, the absence of specific intracellular Aβ_42_ immunoreactivity in this region (Supplementary Figure 4F) suggests that the intracellular 6E10 signal likely represents other Aβ species or APP (Hunter and Brayne, 2017).

It has been hypothesized that oligomeric Aβ species may exert major neurotoxic effects in AD (Lambert et al., 1998). Therefore, we have additionally estimated if the amount of oligomeric Aβ may be altered in 5xFAD-Thy1-Cre mice. We have observed prominent occurrence of 6E10-positive plaques that also displayed immunofluorescence signal derived from the oligomer-specific antibody A11 (Glabe, 2008) in a region of the striatum, which also contained Cre-positive neurons in 5xFAD-Thy1-Cre mice (Supplementary Figure 4G). In plaques from this region, the 6E10-positive Aβ plaque area covered by A11-positive oligomers is indeed lower in 5xFAD-Thy1-Cre mice (**Figures 3I,J**). Moreover, the decrease in the neuronal number observed in hippocampal DG of 5xFAD mice is halted in 5xFAD-Thy1-Cre mice (**Figure 3K**).

Based on these results, we conclude that GCS deletion may lead to decreased deposition of Aβ_42_ and oligomeric Aβ in the 5xFAD mouse model.

### Spatial Memory Is Improved in 5xFAD-Thy1-Cre Mice

**FIGURE 4:**
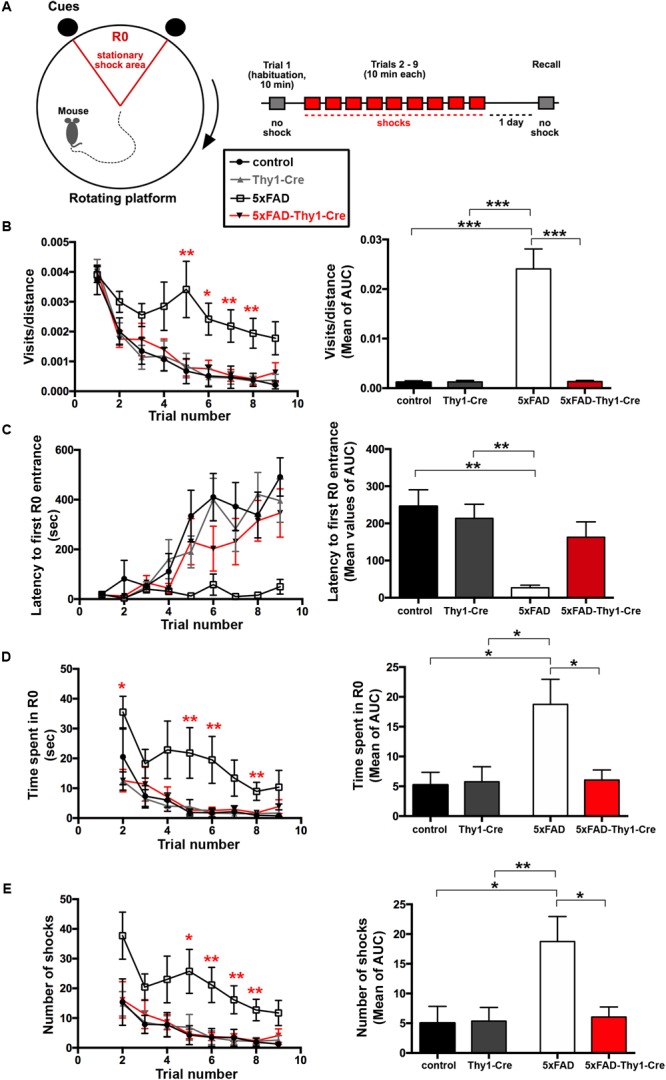
Improved learning and memory in 5xFAD-Thy1-Cre mice (pt. 1). **(A)** Experimental set-up of the active place avoidance test. Mice are placed on a rotating platform and visual cues outline the R0 shock area. Upon entering the R0 area, mice receive mild electric stimuli until leaving the area. An initial trial (habituation) without electric stimulation is followed by eight training trials with electric stimulation. One recall trial without shocks is carried out 24 h later. **(B–E)** Mice were tracked during each trial and the following parameters were monitored: visits/distance **(B)**, latency to first R0 entry **(C)**, time spent in R0 **(D)**, and number of shocks received **(E)**. Learning to avoid R0 during the training trials is improved in 5xFAD-Thy1-Cre mice, compared to 5xFAD mice (*n* = 8 control; 7 5xFAD; 10 Thy1-Cre; 9 5xFAD-Thy1-Cre mice). Additionally, the respective area under the curve (AUC) was determined for each mouse, and resulting means of the four mouse groups are depicted. Means ± SEM. Statistical analysis for all groups and for each time point was performed by one-way ANOVA with Tukey’s test for multiple comparison (confidence interval = 95%). Consequently, in **(B–E)**, the red stars denote the significance for the comparison 5xFAD vs. 5xFAD-Thy1-Cre mice.

Alzheimer’s disease progression is characterized by a decline in cognitive abilities. The spatial memory was assessed by an active place avoidance test, which has the advantage of rapid hippocampus-dependent acquisition and persistent hippocampus-dependent recall (Cimadevilla et al., 2001; Drier et al., 2002; Pastalkova et al., 2006). On a rotating platform, mice learn to avoid the stationary shock area (R0), where they receive mild electric shocks, based on visual cues. The retention of long-term-stored spatial information is assessed 24 h later during a recall phase (Pastalkova et al., 2006; **Figure 4A**).

**FIGURE 5:**
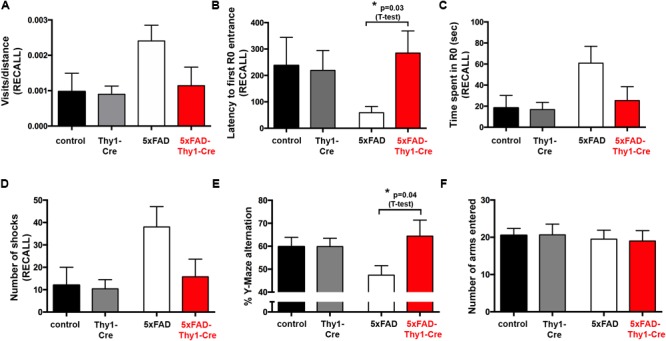
Improved learning and memory in 5xFAD-Thy1-Cre mice (pt. 2). **(A–D)** During a recall trial, the visits/distance **(A)**, latency to first R0 entry **(B)**, time spent in R0 **(C)**, and number of shocks received **(D)** were monitored. As mice do not receive electric shocks during recall, the graph **(D)** monitors the hypothetical shocks mice would have received during their stay in R0 (*n* = 8 control; 7 5xFAD; 10 Thy1-Cre; 9 5xFAD-Thy1-Cre mice). **(E)** 5xFAD-Thy1-Cre mice perform better than 5xFAD mice during a spontaneous Y-maze alternation test (*n* = 17 control; 12 5xFAD; 12 Thy1-Cre; 7 5xFAD-Thy1-Cre mice). **(F)** Activity and explorative behavior in the Y-maze is comparable between all groups of mice. Means ± SEM. Statistical analysis for all four groups was performed by one-way ANOVA with Tukey’s test for multiple comparison (95% confidence interval). An additional statistical comparison of the means of the two primary groups of interest (5xFAD vs. 5xFAD-Thy1-Cre) was performed by a two-tailed Student’s *t*-test and respective *p*-values are depicted in the figure.

In contrast to 5xFAD mice, 5xFAD-Thy1-Cre mice show a significantly lower R0 visiting frequency, which is comparable to non-AD control and Thy1-Cre mice (**Figure 4B** and Supplementary Figure 5A). Likewise, 5xFAD-Thy1-Cre mice enter R0 later (**Figure 4C** and Supplementary Figure 5B), spend less time in R0 (**Figure 4D** and Supplementary Figure 5C), and receive fewer shocks (**Figure 4E** and Supplementary Figure 5D) than 5xFAD mice. Overall, the learning performance and spatial cognitive capacity of 5xFAD-Thy1-Cre mice is comparable to that of control and Thy1-Cre mice during this test. Interestingly, during the recall phase 5xFAD-Thy1-Cre mice also perform better than 5xFAD mice (**Figures 5A–D** and Supplementary Figures 6A–D). Additionally, we have monitored spontaneous Y-maze alternation of 9-month-old mice. In contrast to 5xFAD mice, 5xFAD-Thy1-Cre mice display no Y-maze memory dysfunction (**Figures 5E,F** and Supplementary Figures 6E,F).

These results suggest that spatial memory is improved in 5xFAD-Thy1-Cre mice, when compared to 5xFAD mice.

### Cre-targeted Neurons Are Protected From Aβ-induced Spine Loss

**FIGURE 6:**
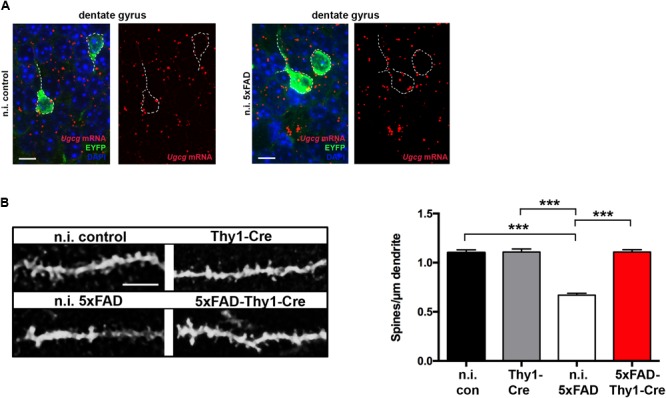
5xFAD-Thy1-Cre mice are protected from spine loss. **(A)** ISH confirms that non-tamoxifen-induced *Ugcg*f/f//Thy1-CreERT2/EYFP mice (n.i. control) and non-induced 5xFAD*/Ugcg*f/f//Thy1-CreERT2/EYFP mice (n.i. 5xFAD) express GCS in EYFP-fluorescent neurons (scale bar = 10 μm). The original brown ISH dots have been converted to red fluorescence, as described in Materials and Methods section. For comparison, the original images are depicted in Supplementary Figure 7A. Non-induced mice received solvent injections without tamoxifen. **(B)** The morphology of EYFP-fluorescent dendrites in the hippocampal dentate gyrus (between lateral 0.96 and 1.2 mm (sagittal)) was analyzed by confocal microscopy and subsequent z-stack reconstruction. A loss of spines is observed in n.i. 5xFAD mice, when compared to n.i. control mice. Intriguingly, spine loss is not observed in 5xFAD-Thy1-Cre mice (*n* = 88 (n.i. control); 122 (n.i. 5xFAD); 156 (Thy1-Cre); 227 (5xFAD-Thy1-Cre) dendritic ROIs from *n* = 3 mice per group; scale bar = 5 μm). Means ± SEM. Statistical analysis for all four groups was performed by one-way ANOVA with Tukey’s test for multiple comparison (95% confidence interval).

Synapse loss is hypothesized to be the strongest anatomical correlate of cognitive impairment in AD (Shankar et al., 2007). In mice, a loss of dendritic spines is observed in advanced AD (Bittner et al., 2010). Hence, we next investigated the spine density. Co-expression of tamoxifen-inducible Cre and tamoxifen-independent EYFP in the same neurons (**Figure 2A**) enabled us to study spines in dendrites of granule cell neurons in the hippocampal DG. In contrast to tamoxifen-induced Thy1-Cre and 5xFAD-Thy1-Cre mice, non-tamoxifen-induced Thy1-Cre without Cre activity (n.i. control mice) and non-tamoxifen-induced 5xFAD-Thy1-Cre mice without Cre activity (n.i. 5xFAD mice) express GCS in EYFP-positive neurons in the hippocampal DG, as indicated by ISH (**Figure 6A** and Supplementary Figure 7A). Spine density of Thy1-Cre mice is indistinguishable from that of n.i. control mice (**Figure 6B**). Moreover, n.i. 5xFAD mice have fewer dendritic spines compared to n.i. control mice, which represents an Aβ-induced spine loss (**Figure 6B**). Strikingly, this Aβ-induced spine loss is not detected in 5xFAD-Thy1-Cre mice (**Figure 6B**).

5xFAD mice display neurodegeneration (Oakley et al., 2006; Herzer et al., 2016) and a concomitant elevation of an abnormal truncated form (p25) of the protein kinase Cdk5 regulatory subunit p35 (Patrick et al., 1999; Oakley et al., 2006). Whereas p25 levels are elevated in 5xFAD mice, they are not changed in 5xFAD-Thy1-Cre mice (Supplementary Figures 7B,C). Moreover, while the p25/p35 ratio is elevated in 5xFAD mice, it is also slightly increased in 5xFAD-Thy1-Cre mice (Supplementary Figure 7D).

Brain insulin signaling is neuroprotective (Plum et al., 2005; De Felice, 2013). In AD, a loss of neuronal IR contributes to the disruption of synaptic function and neurodegeneration (De Felice, 2013). We have previously shown that *Ugcg* deletion protects cortical neurons of 5xFAD mice from the loss of IR pertinent to Aβ pathology (Herzer et al., 2016). A western blot shows that 5xFAD mice applied in this study also display a decrease in IR in hippocampal tissue (Supplementary Figure 7E). In line with the data from our previous work (Herzer et al., 2016), 5xFAD-Thy1-Cre mice are protected from the loss of hippocampal IR (Supplementary Figure 7E).

### Altered Astrocyte Morphology and Pro-inflammatory Cytokine Expression on 5xFAD-Thy1-Cre Mice

**FIGURE 7:**
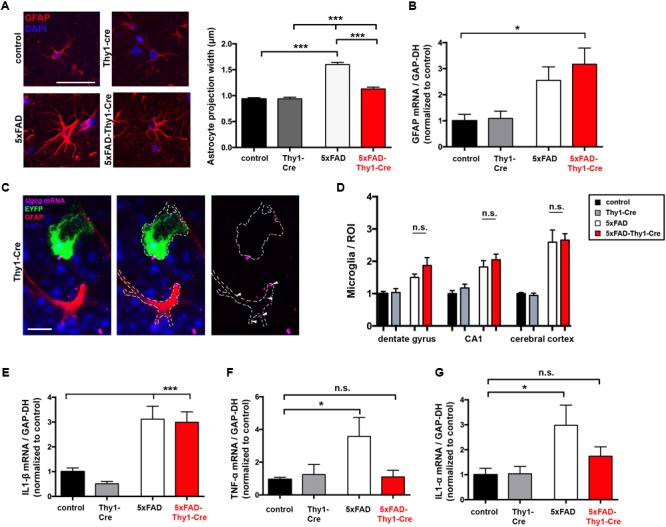
Thickening of astrocytic processes and expression of TNF-α and IL1-α are less pronounced in 5xFAD-Thy1-Cre mice. **(A)** A glial fibrillary acidic protein (GFAP) staining of astrocytes in the hippocampal dentate gyrus (between lateral 0.96 and 1.2 mm (sagittal)) depicts astrocytic processes. Astrocytic process width is significantly elevated in 5xFAD mice. In comparison to 5xFAD mice, the thickening of astrocytic processes is less pronounced in 5xFAD-Thy1-Cre mice (*n* = 348 (control); 378 (5xFAD); 320 (Thy1-Cre); 312 (5xFAD-Thy1-Cre) projections derived from 73 control, 88 5xFAD, 68 Thy1-Cre, 74 5xFAD-Thy1-Cre cells (three mice per group); scale bar = 50 μm). **(B)** A quantitative mRNA expression analysis from hippocampal tissue shows that GFAP expression is elevated in both 5xFAD and 5xFAD-Thy1-Cre mice. Expression levels have been normalized to glyceraldehyde-3-phosphate dehydrogenase (GAPDH) expression (*n* = 7 control; 5 5xFAD; 9 Thy1-Cre; 8 5xFAD-Thy1-Cre mice). **(C)** An ISH shows that glial cells of Thy1-Cre mice express *Ugcg* mRNA and that they are not targeted by GCS deletion (scale bar = 10 μm). The original brown ISH dots have been converted to purple fluorescence, as described in the respective Materials and Methods section. For comparison, the original image is depicted in Supplementary Figure 8A. **(D)** The number of ionized calcium binding adaptor molecule 1 (Iba1)-positive microglia is elevated in dentate gyrus, CA1, and cerebral cortex of both 5xFAD and 5xFAD-Thy1-Cre mice (*n* = 7 (control, Thy1-Cre, 5xFAD) and 8 (5xFAD-Thy1-Cre) sections (ROIs) derived from 5 control or 5xFAD-Thy1-Cre and 4 5xFAD or Thy1-Cre mice). **(E)** A quantitative mRNA expression analysis from hippocampal tissue shows that IL1-β expression is significantly elevated in both 5xFAD and 5xFAD-Thy1-Cre mice. Expression levels have been normalized to GAPDH expression (*n* = 7 control; 5 5xFAD; 9 Thy1-Cre; 8 5xFAD-Thy1-Cre mice). **(F,G)** Quantitative mRNA expression analyses from hippocampal tissue show that tumor necrosis factor-α (TNF-α) and interleukin 1-α (IL1-α) expression levels are significantly elevated in 5xFAD mice. However, the expression of these cytokines is not significantly elevated in 5xFAD-Thy1-Cre mice. Expression levels have been normalized to GAPDH expression (*n* = 7 control, 5 5xFAD, 9 Thy1-Cre, 8 5xFAD-Thy1-Cre mice). Means ± SEM. Statistical analysis for all four groups was performed by one-way ANOVA with Tukey’s test for multiple comparison (95% confidence interval). Means ± SEM.

Neurodegeneration and spine loss in AD are accompanied by a massive activation of astrocytes (reactive astrocytosis) and microglia, followed by an increase in pro-inflammatory cytokine secretion (e.g., interleukin (IL)-1α and tumor necrosis factor (TNF)-α) (Krstic and Knuesel, 2013). In AD mice, astrocytic activation is characterized by a pronounced thickening of astrocytic processes (Doméné et al., 2016). Nine-month-old 5xFAD mice display enlarged astrocytic processes within the hippocampal DG (**Figure 7A**). However, astrocytic processes are significantly less enlarged in 5xFAD-Thy1-Cre mice (**Figure 7A**), whereas glial fibrillary acidic protein (GFAP) expression *per se* does not differ between 5xFAD and 5xFAD-Thy1-Cre mice (**Figure 7B**). An ISH confirms that *Ugcg* deletion in 5xFAD-Thy1-Cre mice is restricted to targeted neurons, whereas the non-targeted astrocytes retain *Ugcg* expression (**Figure 7C** and Supplementary Figure 8A).

It has been recently suggested that two different types of reactive astrocytes may be distinguished (Liddelow et al., 2017). On the one hand, harmful astrocytes (termed A1 astrocytes) with up-regulated classical complement cascade genes contribute to synaptotoxicity (Liddelow et al., 2017). On the other hand, so-called A2 astrocytes exert neurotrophic actions and are thus considered to be rather protective (Liddelow et al., 2017). Activated microglia play an important role in inducing A1 astrocytes. Interestingly, the number of ionized calcium-binding-adaptor-molecule-1 (Iba-1)-positive microglia is not altered between 5xFAD and 5xFAD-Thy1-Cre mice in cerebral cortex, hippocampal CA1, and hippocampal DG (**Figure 7D** and Supplementary Figure 8B). Moreover, *Il1-β* mRNA is elevated in both 5xFAD and 5xFAD-Thy1-Cre mice (**Figure 7E**). Importantly, however, the levels of *Tnf-α* and *Il1-α* mRNA, which are suggested to induce synaptotoxic A1 astrocytes (Liddelow et al., 2017), are decreased in 5xFAD-Thy1-Cre mice (**Figures 7F,G**).

These results suggest that astrocyte activation and microgliosis take place in 5xFAD and 5xFAD-Thy1-Cre mice. However, this work shows that the cognitive capacity is improved and synapses in hippocampal dendrites are protected in 5xFAD-Thy1-Cre mice. Thus, we hypothesize that the altered astrocyte morphology in conjunction with a decrease in the pro-inflammatory cytokine TNF-α and IL1-α expression may contribute to the beneficial effects that GCS deletion exerts on the course of disease in 5xFAD-Thy1-Cre mice.

## Discussion

Complex glycosphingolipids have emerged as novel targets in neurodegenerative diseases (Ariga et al., 2008; Herzer et al., 2016; Henriques et al., 2017). Recently, we reported that pharmacological GCS inhibition protects cultured hippocampal neurons from soluble oligomeric-Aβ_1-42_-induced neurotoxicity and loss of surface IR, presumably via down-regulation of caveolin-1 (Herzer et al., 2016). Moreover, we provided evidence that inducible GCS deletion in adult forebrain neurons supports neuronal survival and stabilizes IR levels in 5xFAD mice (Herzer et al., 2016). Our current work now indicates that GCS deletion in adult forebrain neuronal subsets improves spatial memory in 5xFAD mice and may counteract Aβ-induced spine loss in the hippocampal DG.

In our previous study (Herzer et al., 2016), we presented data from *Ugcg*f/f//CamKCreERT2 mice that, amongst others, display strong Cre activity in the hypothalamus, which causes an obese phenotype (Nordström et al., 2013; Herzer et al., 2015). For the current study, we utilized *Ugcg*f/f//Thy1-CreERT2/EYFP mice (Thy1-Cre mice), which (i) express EYFP in Cre-targeted neurons and (ii) display normal body weight. EYFP fluorescence enabled us to analyze dendritic spines in neurons targeted by the construct. Since the hypothalamus of Thy1-Cre mice is not targeted by Cre activity (Young et al., 2008), these mice do not display obesity. In line with the findings from Young et al. (2008), hippocampal neurons of Thy1-Cre mice are prominently targeted by Cre activity. In combination with normal body weight, this makes theses mice very suitable for studying spatial memory.

Glucosylceramide synthase-derived gangliosides are important components of dynamic membrane microdomains, which are widely acknowledged to modulate transmembrane receptor function (Mutoh et al., 1995; Kabayama et al., 2007; Nordström et al., 2013; Herzer et al., 2015, 2016) and membrane processes, such as endocytosis (Ewers et al., 2010). Moreover, gangliosides are essential for initial brain maturation (Jennemann et al., 2005). Consequently, conditional *Ugcg* deletion in the whole brain, comprising neurons and glial cells, under the control of the nestin promoter results in loss of Purkinje cells and an abnormal behavior (Yamashita et al., 2005). These studies reflect the essential importance of ganglioside biosynthesis in the developing brain. However, it is important to distinguish these findings from the approach applied in the current study, namely the tamoxifen-inducible *Ugcg* deletion strictly confined to subsets of adult forebrain neurons. It is important to mention that GCS deletion in adult neurons does not lead to any ultra-structural abnormalities in cell organelles and membrane nor changes basic electrophysiological properties (Nordström et al., 2013). Moreover, constitutive GCS deletion in the brain does not increase the number of apoptotic neurons (Jennemann et al., 2005). Thus, the protective effects observed in our 5xFAD-Thy1-Cre mice are not in conflict with the studies showing that GCS deletion in the embryonic brain is indeed deleterious.

Alterations in ganglioside biosynthesis have been described in models of AD (Ariga et al., 2008). More specifically, the ganglioside precursor lactosylceramide drives inflammatory processes in a mouse model of multiple sclerosis and is suggested to contribute to other neurodegenerative disorders (Mayo et al., 2014). Thus, these reports emphasize that GCS is an important novel target against neurodegenerative diseases. In support of this suggestion, a previous study has reported that elimination of GD3 synthase responsible for the biosynthesis of b- and c-series gangliosides including two of the four major brain gangliosides GD1b and GT1b resulted in decreased Aβ plaque formation and restoration of cognitive function in double-transgenic APP/PSEN1 mice (Bernardo et al., 2009), potentially in part due to an increased biosynthesis of the otherwise minor ganglioside GT1aα in APP/PSEN1/GD3S-/- mice (Ariga et al., 2013). Those results are in line with our finding that ganglioside depletion restores memory function in AD mouse models. They furthermore support our observation that the deposition of Aβ_40_, Aβ_42_, and oligomeric Aβ is decreased in the hippocampus of 5xFAD-Thy1-Cre mice, where Cre-targeted neurons are present. However, we surprisingly do not detect any decrease in 6E10-positive plaque formation, neither in 5xFAD-Thy1-Cre mice nor in the 5xFAD-Ugcgf/f//CamKCreERT2 mouse model analyzed in our previous work (Herzer et al., 2016). Whereas the morphology and numbers of 6E10-stained plaques are not altered (Supplementary Figures 3A–D), the MALDI analysis reveals that the intensity of specific Aβ_40_ and Aβ_42_ signals is slightly reduced in 5xFAD-Thy1-Cre mice, also confirming the decreased Aβ_42_ deposition detected by immunohistochemistry (**Figures 3A–D**). Of note, the 6E10 antibody tends to have a certain cross-reactivity with APP and APP cleavage products apart from Aβ (Hunter and Brayne, 2017). Therefore, the 6E10 signal likely does not distinguish between Aβ_42_, Aβ_40_, APP, and potentially other Aβ species, neither in plaques nor intracellular. Moreover, the five mutations present in 5xFAD mice elicit a vast overexpression of aggregation-prone Aβ species, starting as early as at the age of 2 months (Oakley et al., 2006). Even though GCS deletion obviously does not interfere with APP and overall Aβ generation, as detected by 6E10, we conclude that deposition of oligomeric Aβ, as well as of aggregation-prone Aβ_42_ may indeed be counteracted by GCS deletion in nearby neurons. Oligomeric Aβ species are hypothesized to exert major neurotoxic effects on neurons in AD (Lambert et al., 1998), and also significantly interfere with synaptic function (De Felice et al., 2009). Thus, a decrease in the deposition of these specific Aβ species likely contributes to the improvement of memory and spine density observed in 5xFAD-Thy1-Cre mice.

Various studies have analyzed connections between sphingolipid metabolism and Aβ generation. Gangliosides GM3 and GD3 have been suggested to decrease or increase amyloidogenic processing of APP, respectively, while Aβ itself is supposed to inhibit GD3 synthase (Grimm et al., 2012). In the current work, we have observed significantly increased GM3 levels in cortex and hippocampus of 5xFAD mice (**Figures 1B,C**). If this increase in GM3 is confined to neurons, an abolition of GM3 may therefore potentially counteract inhibitory effects on Aβ processing. However, future studies would need to clarify if increased GM3 in 5xFAD mice is indeed confined to neurons or, since GM3 is a major ganglioside expressed in astrocytes (Sbaschnig-Agler et al., 1988; Murakami et al., 1999), if GM3 levels may rather reflect the concomitant astrogliosis.

The major neuronal ganglioside GM1 has been suggested to act as a membranous “seed”, promoting binding and fibrillogenesis of monomeric Aβ, which clearly demonstrates the potential of neuronal gangliosides to promote AD pathology (Yanagisawa et al., 1995). However, it has to be noted that the experiments directly supporting the “seeding” theory were mainly conducted in *in situ* binding assays rather than in a mouse model (Yanagisawa et al., 1995), and may therefore not be directly comparable to the situation in our 5xFAD mouse model. On the other hand, central administration of GM1 enhanced the amyloid burden in mice overexpressing Aβ (Matsuoka et al., 2003), even though the contribution of the other major neuronal ganglioside species to Aβ pathology have not been outlined. Moreover, we have previously reported that GCS inhibition does not hamper binding of Aβ oligomers to ganglioside-deficient cultured neurons, suggesting that Aβ species apart from monomers may not depend on membrane gangliosides as “seeds” (Herzer et al., 2016). Of note, residual non-targeted neurons still express GM1, which in turn may act as a potential “seed” for Aβ fibril formation in our mice. These aspects may contribute to the observation that the overall 6E10-positive plaque load is not reduced in 5xFAD-Thy1-Cre mice.

The mass spectrometry analysis of other important brain lipids in hippocampal tissue showed that ceramide and lactosylceramide were slightly elevated in 5xFAD and 5xFAD-Thy1-Cre mice. Contrary to 5xFAD-Thy1-Cre mice, sphingomyelin was slightly, but not significantly reduced in 5xFAD mice. This effect is not surprising, as it has been shown in humans that sphingomyelin levels drop in AD due to an increased activity of sphingomyelinase (He et al., 2010). The slight decrease in hexosylceramides, including galactosylceramide and glucosylceramide, in Thy1-Cre and 5xFAD-Thy1-Cre mice much likely reflects the loss of glucosylceramide in Cre-targeted neurons.

In line with previous reports (Oakley et al., 2006; Leinenga and Götz, 2015), learning and memory retention is impaired in our 5xFAD mice during the active place avoidance test. Moreover, 5xFAD mice display a lower spontaneous Y-maze alternation (Oakley et al., 2006), which we could confirm. A key finding of our study is the fact that the cognitive function of 5xFAD-Thy1-Cre mice is improved in both tests, compared to 5xFAD mice. Moreover, 5xFAD-Thy1-Cre mice show a better memory retention, even though the amount of Cre-targeted neurons in the hippocampus of 5xFAD-Thy1-Cre mice is variable and the targeting rate in the region of the dorsal hippocampus that we have analyzed is moderate. However, it has to be noted that the transgenes in 5xFAD mice and the targeting construct in Thy1-Cre mice are both expressed under the control of the *Thy1* promoter. Thus, all Cre-targeted neurons harbor 5xFAD mutations. Our observation that 5xFAD-Thy1-Cre mice perform better than 5xFAD mice in the cognitive assays speaks in favor of the assumption that the beneficial effects of GCS deletion in the numbers of Cre-targeted neurons present seem to be sufficient for memory improvement. Indeed, the inhibition of ganglioside biosynthesis does not solely impact Cre-targeted neurons, but also interferes with cell–cell communication (Lopez and Schnaar, 2009) and, through its effects on endo- and exocytosis (Ewers et al., 2010), potentially with neuropeptide release. Thus, neighboring neuronal and non-neuronal cells that have not been originally targeted can potentially be influenced by GCS-deficient neurons. In fact, the observed beneficial effects on pro-inflammatory cytokine levels released by non-targeted glial cells in 5xFAD-Thy1-Cre mice further support this hypothesis. However, further studies are needed to clarify how neuronal GCS deletion may influence non-targeted cells.

In conjunction with the cognitive decline, a decrease in the dendritic spine density in AD-affected brain regions has been reported in AD mice (Bittner et al., 2010). In case of n.i. 5xFAD and 5xFAD-Thy1-Cre mice, we have analyzed spines in neuronal processes that were located adjacent to plaques (up to 30 μm distance). Indeed, aged 5xFAD mice have a lower spine density along neuronal dendrites of the hippocampal DG than control mice (**Figure 6B**), which goes in line with a previous study reporting profound spine loss (∼50%) in processes adjacent (∼20 μm) to amyloid plaques (Spires et al., 2005). Strikingly, however, Cre-targeted neurons in 5xFAD-Thy1-Cre mice are protected from spine loss.

Appropriate neuronal insulin sensitivity and signaling are vital for the maintenance and formation of new synapses, and the loss of neuronal IR is thought to significantly contribute to Aβ-induced cognitive decline (De Felice, 2013). In our previous study, we demonstrated that GCS inhibition can stabilize neuronal IR levels and signaling (Herzer et al., 2016). Since 5xFAD-Thy1-Cre mice are also protected from the loss of IR in the hippocampus (Supplementary Figure 7E), we suggest that a stabilization of IR in targeted neurons might also contribute to the maintenance of spines in these mice. However, we would like to point out that a loss of neuronal gangliosides potentially alters additional processes, such as neurotransmitter release (Zitman et al., 2010) and intercellular interactions with glial cells. Therefore, delineating further molecular mechanisms that explain how a decrease in neuronal gangliosides could possibly protect dendritic spines in AD remains a promising target for future studies.

The neurodegeneration marker p25 is an abnormal truncated form of the regulatory subunit p35 of the protein kinase Cdk5 (Patrick et al., 1999; Oakley et al., 2006). Even though studies have reported both elevated (Patrick et al., 1999) and unchanged (Yoo and Lubec, 2001) p25 levels in human AD brains, p25 is indicative for neurodegeneration in mice (Ahlijanian et al., 2000). Compared to 5xFAD mice, the p25 levels are slightly decreased in hippocampal tissue of 5xFAD-Thy1-Cre mice. Moreover, we observe that the loss of hippocampal DG neurons observed in 5xFAD mice is halted in 5xFAD-Thy1-Cre mice (**Figure 3K**). These results are in conjunction with our previous data showing that neurodegeneration in cerebral cortex and p25 increase are counteracted in 5xFAD-CamKCre mice (Herzer et al., 2016). Thus, we postulate that inhibiting ganglioside biosynthesis may protect adult neurons from neurodegeneration that is pertinent to AD.

In recent years, refined hypotheses of the initiation and progression of AD have emerged. Importantly, glial cells are regarded as central contributors to neuroinflammation in AD (Hensley, 2010; Krstic and Knuesel, 2013). Prominent astrogliosis and microgliosis with increased GFAP and Iba1 expression are common hallmarks of AD-related neuroinflammation, which contributes to the cognitive decline in AD (Krstic and Knuesel, 2013). Therefore, the elevated GFAP expression and number of Iba1-positive microglia in both 5xFAD and 5xFAD-Thy1-Cre mice was initially surprising. However, it is important to note that astrocyte activation with GFAP increase *per se* cannot be considered to be “detrimental-only” in AD, as AD pathogenesis worsens in GFAP-deficient mice (Kraft et al., 2013). Rather, astrocytes are suggested to clear neurotoxic Aβ from the brain and may thereby protect neurons (Xiao et al., 2014). Thus, the contribution of activated astrocytes to AD pathology has been a topic of intense research. Recently, Liddelow et al. (2017) described distinct subpopulations of activated astrocytes: neurotoxic A1 astrocytes and neuroprotective A2 astrocytes. Liddelow et al. (2017) showed that induction of A1 astrocytes requires the pro-inflammatory cytokines IL1-α and TNF-α. We have now found that the expression of IL1-α and TNF-α tends to be lower in 5xFAD-Thy1-Cre mice, compared to 5xFAD mice. However, we are aware that the definite nature of the astrocytes needs to be determined in future studies. Importantly, the induction of A1 and A2 astrocytes by pro-inflammatory cytokines has only been described in a stimulated *in vitro* cell culture system (Liddelow et al., 2017), and the concept has yet to be transferred to the *in vivo* situation. In general, astrocyte morphology is largely affected under pathological conditions and astrocytes can undergo a dramatic increase in process thickness, which can be quantified by morphometry (Sun et al., 2010). Importantly, the thickening of astrocytic processes is markedly reduced in the hippocampus of 5xFAD-Thy1-Cre mice, compared to 5xFAD mice. Taken together, our data provide hints that certain parameters characteristic for neuroinflammation, such as astrocyte process thickness and the expression of the pro-inflammatory cytokines IL1-α and TNF-α are reduced in 5xFAD-Thy1-Cre mice. As the degree of neuroinflammation was shown to correlate to the clinical severity of AD symptoms (Krstic and Knuesel, 2013), these results speak in favor of the improved spine density and cognitive capacity in 5xFAD-Thy1-Cre mice.

The present study contains potential limitations. Foremost, it was conducted in a mouse model of familial AD, while the majority of patients suffer from sporadic AD (sAD). However, suitable mouse models for sAD still need to be established (Onos et al., 2016). The effects of adult-neuronal GCS deletion on cognition need to be evaluated in sAD animal models upon availability. Another limitation is the application of a genetic adult-neuron-specific GCS deletion model. In order to transfer our results to a potential clinical application, pharmacological GCS inhibitors need to be evaluated for their therapeutic potential, initially in the brains of familial and sporadic AD mouse models. Importantly, a suitable mode of drug application able to cross the blood–brain barrier, and successful targeting of the brain needs to be established in mice. Beneficial effects of pharmacological GCS inhibition, as previously shown in AD models *in vitro* (Herzer et al., 2016), need to be confirmed in AD mice before a translational approach for patients can be developed. Furthermore, our knowledge as to how diminished astrocyte activation contributes to memory improvements in 5xFAD-Thy1-Cre mice is also still limited. Since our model only comprises Cre activity in target neurons, any effects of GCS inhibition in glial cells need to be studied, as pharmacological GCS inhibitors will also target glial cells. Nevertheless, our data, showing long-lasting beneficial effects of cell-specific GCS deletion on cognition, spine density, and astrocyte activation in 5xFAD mice, comprise promising groundwork for the suggested studies.

## Conclusion

We postulate that inhibiting ganglioside biosynthesis in specific subsets of adult forebrain neurons improves cognition and counteracts the loss of dendritic spines in 5xFAD mice. Our work further emphasizes that GCS may emerge as a promising novel therapeutic target for AD and the data warrant further research endeavors as discussed above.

## Author Contributions

VN conceived and supervised the study, analyzed data, and wrote the manuscript. SH conceived experiments, performed experiments, analyzed data, and edited the manuscript. CHa, JvG, BM, and VD performed experiments and analyzed data. CHo and RS provided technical resources.

## Conflict of Interest Statement

The authors declare that the research was conducted in the absence of any commercial or financial relationships that could be construed as a potential conflict of interest.
