# Supplementary material for: Deletion of Specific Sphingolipids in Distinct Neurons Improves Spatial Memory in a Mouse Model of Alzheimer’s Disease
Source: Front Mol Neurosci. 2018 Jun 20;11:206. doi: 10.3389/fnmol.2018.00206 (PMC6019486; doi:10.3389/fnmol.2018.00206)
Supplement: Supplementary file 1 [file Data_Sheet_1.docx]

**Deletion of specific sphingolipids in distinct neurons improves spatial memory in a mouse model of Alzheimer’s disease**

**Supplementary Material**

S. Herzer ^1, 2^, C. Hagan ^1, 3^, J. von Gerichten ^1, 4^, V. Dieterle ^1,2^, B. Munteanu ^5^, R. Sandhoff ^1, 4^, C. Hopf ^5^, and V. Nordström ^1, 2,*^

^1^ Department of Cellular and Molecular Pathology, German Cancer Research Center, 69120 Heidelberg, Germany

^2^ Interdisciplinary Center for Neurosciences, Heidelberg University, 69120 Heidelberg, Germany

^3^ Department of Microbiology, Immunology, and Pathology, Colorado State University, Fort Collins, CO, USA

^4^ Lipid Pathobiochemistry Group, German Cancer Research Center, 69120 Heidelberg, Germany

^5^ Center for Mass Spectrometry (CeMOS), University of Heidelberg and Mannheim University of Applied Sciences, Mannhelm, Germany

* Correspondence: v.nordstroem@dkfz-heidelberg.de; phone: +49 6221 42 4368

**Supplementary Methods**

**Enzyme-linked immunosorbent assay (ELISA)**

Aβ_42_ and Aβ_40_ contents were analyzed in lysates from cerebral cortex of female nine months old mice (*Ugcg* f/f mice (controls), and 5xFAD as well as 5xFAD-Thy1-Cre littermates) by ELISA kits (Cat. no: KHB3481 for Aβ_40_ and KHB3441 for Aβ_42_) according to the manufacturer’s guidelines (Thermo Fisher Scientific). Fresh frozen cortex tissue was processed for ELISA according to the manufacturer’s guidelines. The ELISAs, detecting both natural and synthetic forms of the respective Aβ species, were then used to quantitatively determine Aβ_42_ and Aβ_40_ levels. Detection (colorimetric) occurred in a 96-well plate reader as described in the manufacturer’s guidelines.

**Western blots**

Western blots from tissue lysates were prepared as previously described by us ^17–19^. Total hippocampi were dissected and immediately snap-frozen in liquid N_2_. Tissue lysates were prepared with the Ambion®PARIS^TM^ kit (Thermo Fisher Scientific) according to the manufacturer’s guidelines. Protein concentrations were determined by Bradford assay (Sigma) and equal amounts of protein were loaded onto SDS gels. SDS gel electrophoresis and subsequent transfer to nitrocellulose membrane was performed according to standard procedures. Primary antibodies used for western blot were rabbit-α-p35/p25 (1:500, Santa Cruz Biotechnology), α-GAPDH (1:500 Santa Cruz Biotechnology), and α-IR (1:200, Santa Cruz Biotechnology). Secondary antibodies used were HRP-conjugated swine-α-rabbit (H+L) and HRP-conjugated rabbit-α-mouse (H+L) (1:1000, DAKO). Bands were visualized by chemiluminescence (Amersham) and quantified with ImageJ (National Institutes of Health). Bands were normalized to the respective loading controls.

**Morphometric analysis of amyloid plaques, A11 content, intracellular 6E10, and astrocytic process width**

Paraffin sections from mouse brains (nine months of age) were prepared and subjected to immunostaining as described in the main text. The location of sections was determined with the help of the Paxinos Brain Atlas. The number of sections and individual mice is indicated in the respective figure legends. Morphometric analyses of 6E10 plaques were carried out with ImageJ (NIH). For 6E10 plaque morphology, all figures were equally adjusted in threshold (ImageJ) in order to minimize residual non-plaque- and intracellular 6E10 signal.

**Mass spectrometry analysis of sphingolipids**

Aliquots corresponding to 0.1 mg protein/mL were mixed with internal lipid standard (ISD, Supplementary Fig. 1A) for analysis by LC-MS/MS using an Aquity I-class UPLC and a Xevo TQ-S “triple-quadrupole” instrument, both from Waters. Using a CSH C18 column (2.1 mm x 100 mm; 1.7 µm, Waters), sphingolipids were measured in positive mode with a gradient between 57% solvent A (50% methanol) and 100 % solvent B (99 % isopropanol, 1% methanol), both containing 10 mM ammonium formate, 0.5% formic acid and 5 µM citrate as additives. Sphingolipids were analyzed with MS/MS-transitions for the protonated molecular ion and the corresponding loss of water by multireaction monitoring (MRM) at optimized collision (Supplementary Fig. 1A). Transitions reflect by majority sphingolipid species with d18:1 long chain base (C18-sphingosine) and C16 to C24 fatty acyl chain length, as C18 sphingosine is the dominant sphingoid base. Two-way Anova was conducted with GraphPad Prism (GraphPad Software, San Diego, CA) and P-value ≤ 0.05 was considered to be statistically significant. Data were presented as the mean ±SEM.

**Supplementary Figure 1. Mass spectrometry parameters**

(A) LC-MS/MS - MRM transitions and collision energy for gangliosides and other sphingolipids (SL). (B) LC-MS/MS – extracted ion chromatograms for gangliosides of the human ganglioside external standard.

**Supplementary Figure 2. Characterization of *Ugcg*f/f//Thy1-CreERT2//EYFP mice**

(A) Percentages of NeuN/EYFP double-positive Cre-targeted neurons in hippocampal dentate gyrus and CA1 determined in sagittal sections located between lateral 0.96 mm and 1.2 mm. Neurons have been counted in 20 sections derived from 5 individual 5xFAD-Thy1-Cre mice. (B) *Ugcg*f/+//Thy1-CreERT2//EYFP mice were crossed with R26R RosaLacZ reporter mice. Cryosections were prepared and stained for LacZ activity (X-Gal) in Cre-targeted neurons. The X-Gal staining reveals that Cre is active in subsets of neurons in the hippocampal dentate gyrus and CA1 region (scale bar = 200µm). (C) Original images for Fig. 2C. An *in situ* hybridization (ISH) of tamoxifen-induced *Ugcg*f/f//Thy1-CreERT2/EYFP mice (Thy1-Cre mice) confirms that Cre-targeted neurons are devoid of GCS expression. The original brown ISH dots are depicted (scale bar = 10µm). (D) ISH of Thy1-Cre mouse with the negative control probe DAPB (ACD) confirms the specificity of the signal. (E) Lipid levels in hippocampal tissue of mice were analyzed by liquid chromatography-coupled tandem mass spectrometry (hexosylceramides (HexCer) comprising glucosylceramide and galactosylceramide, ceramide (Cer), lactosylceramide (LacCer), sphingomyelin (SM); n = 6 hippocampal hemispheres from n = 3 8-months old mice per group). (F) 40-weeks old Thy1-Cre mice do not develop obesity (n=4 mice). Means ± SEM; unpaired two-tailed Student’s T-test; (#) if *P* ≤0.1. Data in (E) were analyzed by one-way ANOVA with Tukey’s test for multiple comparison (95% confidence interval).

**Supplementary Figure 3. 6E10-positive plaque morphometry**

A) Hippocampal sections (between lateral 0.96 mm and 1.2 mm (sagittal)) were stained with 6E10 in order to visualize amyloid plaques (sb = subiculum, DG = dentate gyrus, scale bar = 300µm). Morphometry of these sections shows that (B) the size and (C) the number of 6E10-positive plaques, as well as (D) the 6E10 plaque load does not differ between 5xFAD and 5xFAD-Thy1-Cre mice. (n = 5 sections derived from 5 mice each; 3924 (5xFAD) and 4107 (5xFAD-Thy1-Cre) plaques were analyzed in total). Statistics for were performed by unpaired two-tailed Student’s T-test. Means ± SEM.

**Supplementary Figure 4. Amyloid-β metabolism in 5xFAD and 5xFAD-Thy1-Cre mice**

(A) Overview MALDI images depicting Aβ_40_ and Aβ_42_ signals, as well as hematoxylin & eosin stainings of brain sections (scale bars = 2mm). (B) An ELISA shows that Aβ_42_ levels in cortical tissue of 5xFAD and 5xFAD-Thy1-Cre mice are comparable (n= 6 control, 8 5xFAD, 9 5xFAD-Thy1-Cre mice). (C) An ELISA shows that Aβ_40_ levels in cortical tissue of 5xFAD and 5xFAD-Thy1-Cre mice are comparable (n= 6 control, 8 5xFAD, 9 5xFAD-Thy1-Cre mice). (D) Intracellular 6E10 signals have been visualized by immunohistochemistry in hippocampal CA1 of sagittal sections located between lateral 0.96 mm and 1.2 mm (scale bar = 10µm). (E) No difference in intracellular 6E10 signal intensity is observed between 5xFAD and 5xFAD-Thy1-Cre mice (n = 145 (5xFAD) and 197 (5xFAD-Thy1-Cre) cells in the hippocampal CA1 region in sagittal sections between lateral 0.96 mm and 1.2 mm derived from 5 individual mice each). (F) An immunohistochemistry shows that intracellular signal of a specific Aβ_42_ antibody is absent in the CA1 region of mice depicted in (E) (scale bar = 50µm). (G) Immunofluorescence depicts the area in the striatum (lateral 0.96 mm and 1.2 mm (sagittal)) of 5xFAD-Thy1-Cre mice, where 6E10 plaques were morphometrically analyzed for A11 content (related to Fig. 3I-J). White arrowheads outline EYFP-and NeuN-positive Cre-targeted neurons (scale bar = 50µm). Means ± SEM. Statistical analysis was performed by one-way-ANOVA with Tukey’s test for multiple comparison (95% confidence interval).

**Supplementary Figure 5. Improved learning and memory in 5xFAD-Thy1-Cre mice (pt. 1)**

Scatter plots depicting individual data points of the data shown in Figure 4 (B) – (E). Mice were tracked during each trial of the active place avoidance test and the following parameters were monitored: visits/distance (A), latency to first R0 entry (B), time spent in R0 (C), and number of shocks received (D). Learning to avoid R0 during the training trials is improved in 5xFAD-Thy1-Cre mice, compared to 5xFAD mice. Means ± SEM are additionally depicted.

**Supplementary Figure 6. Improved learning and memory in 5xFAD-Thy1-Cre mice (pt. 2)**

Scatter plots depicting individual data points of the data shown in Figure 5 (A) – (F). During a recall trial, the visits/distance (A), latency to first R0 entry (B), time spent in R0 (C), and number of shocks received (D) were monitored. As mice do not receive electric shocks during recall, the graph (D) monitors the hypothetical shocks mice would have received during their stay in R0. The recall trial shows that memory retention is improved in 5xFAD-Thy1-Cre mice, when compared to that of 5xFAD mice. (E) 5xFAD-Thy1-Cre mice perform better than 5xFAD mice during a spontaneous Y-maze alternation test. (F) Activity and explorative behavior in the Y-maze is comparable between all groups of mice. Means ± SEM are additionally depicted.

**Supplementary Figure 7. Original *in situ* hybridization pictures from Figure 6A, lower p25 levels and normal IR levels in 5xFAD-Thy1-Cre mice**

(A) ISH confirms that non-tamoxifen-induced *Ugcg*f/f//Thy1-CreERT2/EYFP mice (n.i. control) and non-induced 5xFAD*/Ugcg*f/f//Thy1-CreERT2/EYFP mice (n.i. 5xFAD) express GCS in EYFP-fluorescent neurons (scale bars = 10µm). The original brown ISH dots corresponding to Fig. 6A are depicted in an overlay picture. Non-induced mice received solvent injections. (B) A western blot shows that the neurodegeneration marker p25 (C) is increased in hippocampal tissue of 5xFAD mice, but not in 5xFAD-Thy1-Cre mice (n= 5 control, 6 5xFAD, 10 Thy1-Cre, 11 5xFAD-Thy1-Cre mice). Moreover, the p25/p35 ratio is shown (D). (E) The insulin receptor (IR) reduction in 5xFAD mice is not observed in 5xFAD-Thy1-Cre mice (n = 6 control, 7 5xFAD, 10 Thy1-Cre and 5xFAD-Thy1-Cre mice). Means ± SEM. Statistical analysis for all four groups was performed by one-way ANOVA with Tukey’s test for multiple comparison (95% confidence interval). An additional statistical comparison of the means of two primary groups of interest was performed by an unpaired two-tailed Student’s T-test and the respective p-values are depicted in the figure.

**Supplementary Figure 8. Microglia in hippocampal dentate gyrus, CA1, and cerebral cortex**

(A) An ISH shows that glial cells of Thy1-Cre mice express *Ugcg* mRNA and that they are not targeted by GCS deletion (scale bar = 10µm). An overlay picture with the original brown ISH dots corresponding to Fig. 7C is depicted. (B) An Iba1 immunofluorescence reveals microglia of the hippocampal dentate gyrus and CA1 region and the cerebral cortex in sagittal sections located between lateral 0.96 mm and 1.2 mm. A quantification of microglial numbers in control, Cre, 5xFAD, and 5xFAD-Thy1-Cre mice is shown in Fig. 7D. Scale bar=50µm.
