# Supplementary figures and images for: Deletion of Specific Sphingolipids in Distinct Neurons Improves Spatial Memory in a Mouse Model of Alzheimer’s Disease
Source: Front Mol Neurosci. 2018 Jun 20;11:206. doi: 10.3389/fnmol.2018.00206 (PMC6019486; doi:10.3389/fnmol.2018.00206)

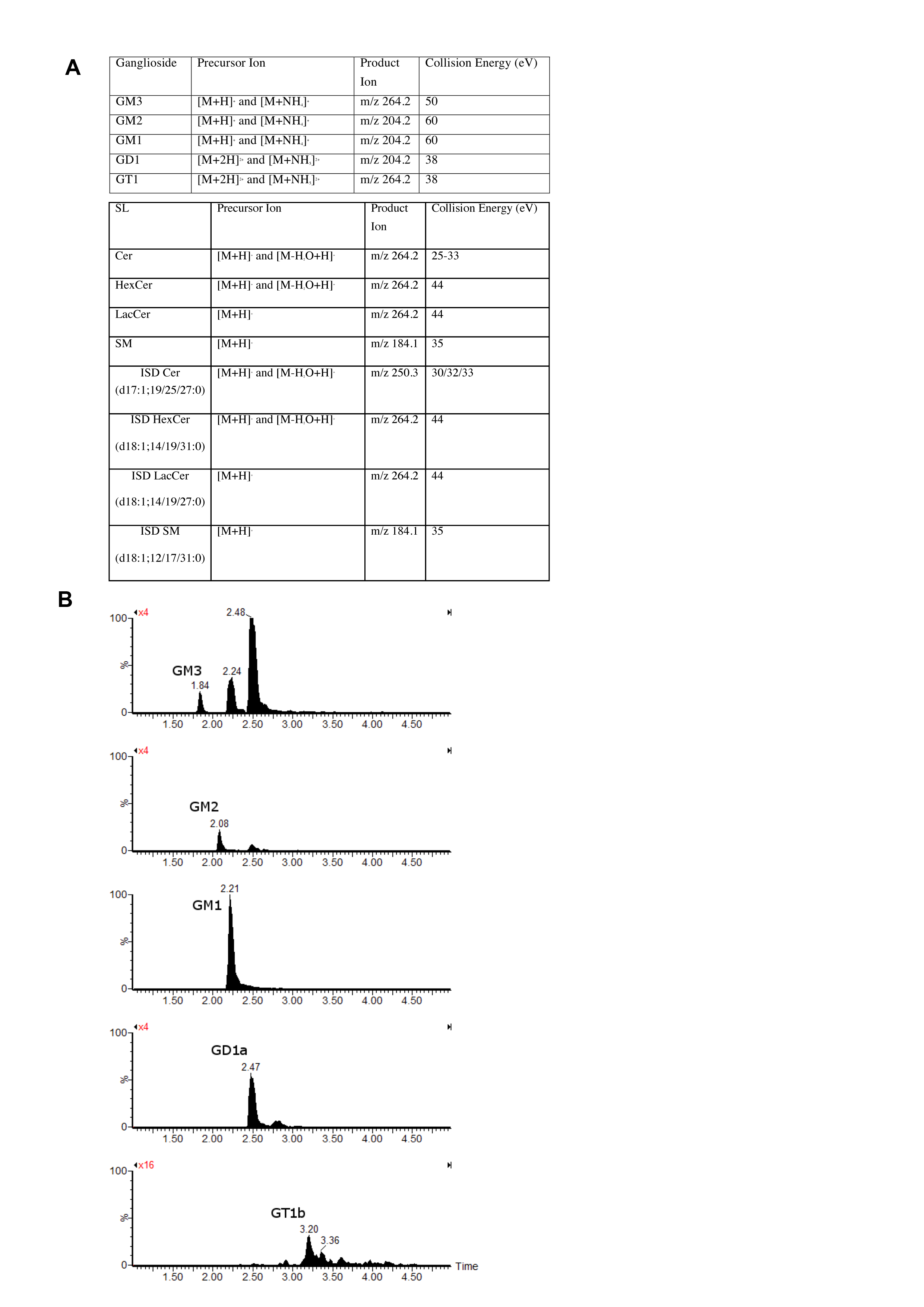

Supplement: Supplementary file 2 [file Image_1.TIF]

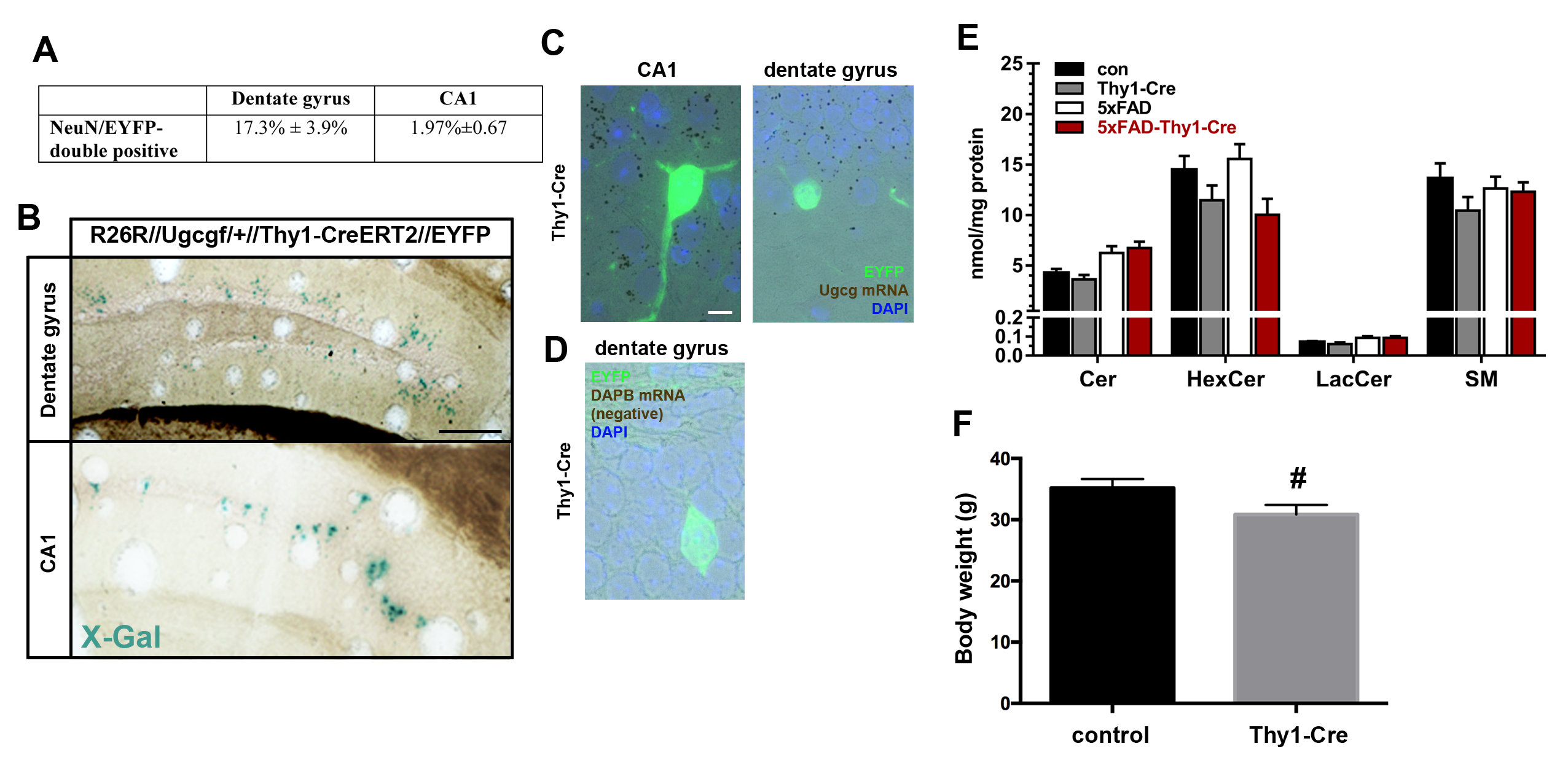

Supplement: Supplementary file 3 [file Image_2.TIF]

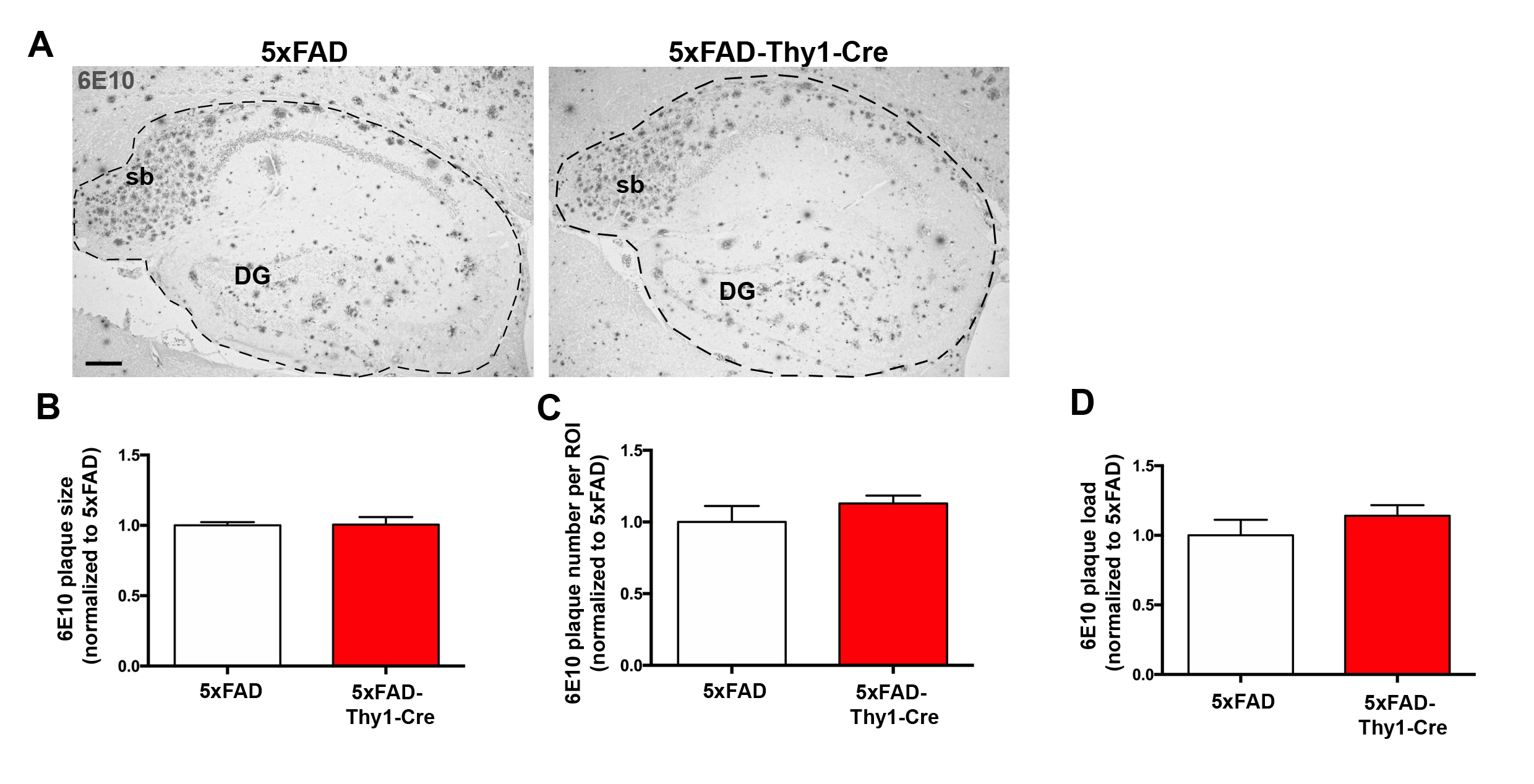

Supplement: Supplementary file 4 [file Image_3.TIF]

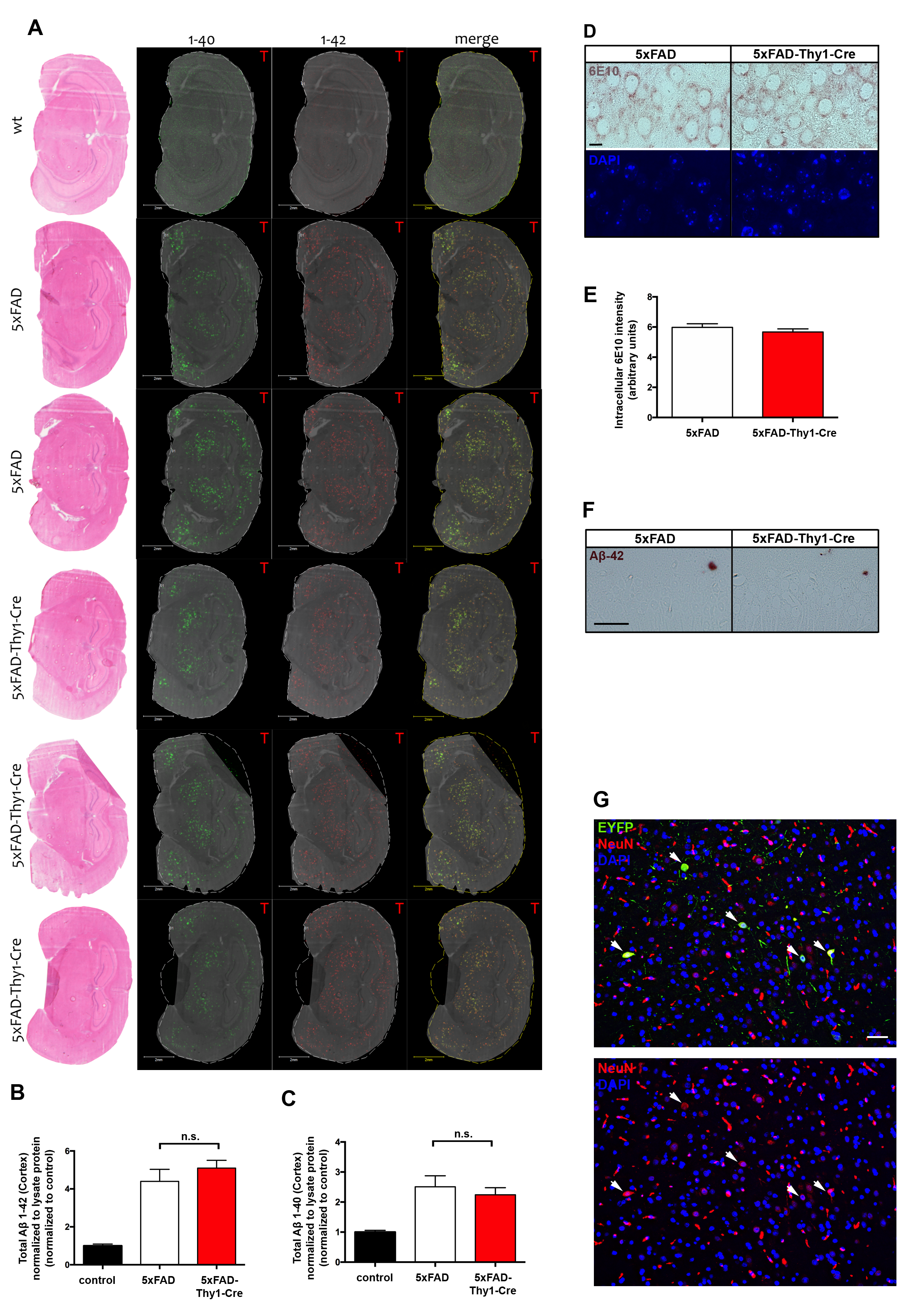

Supplement: Supplementary file 5 [file Image_4.TIF]

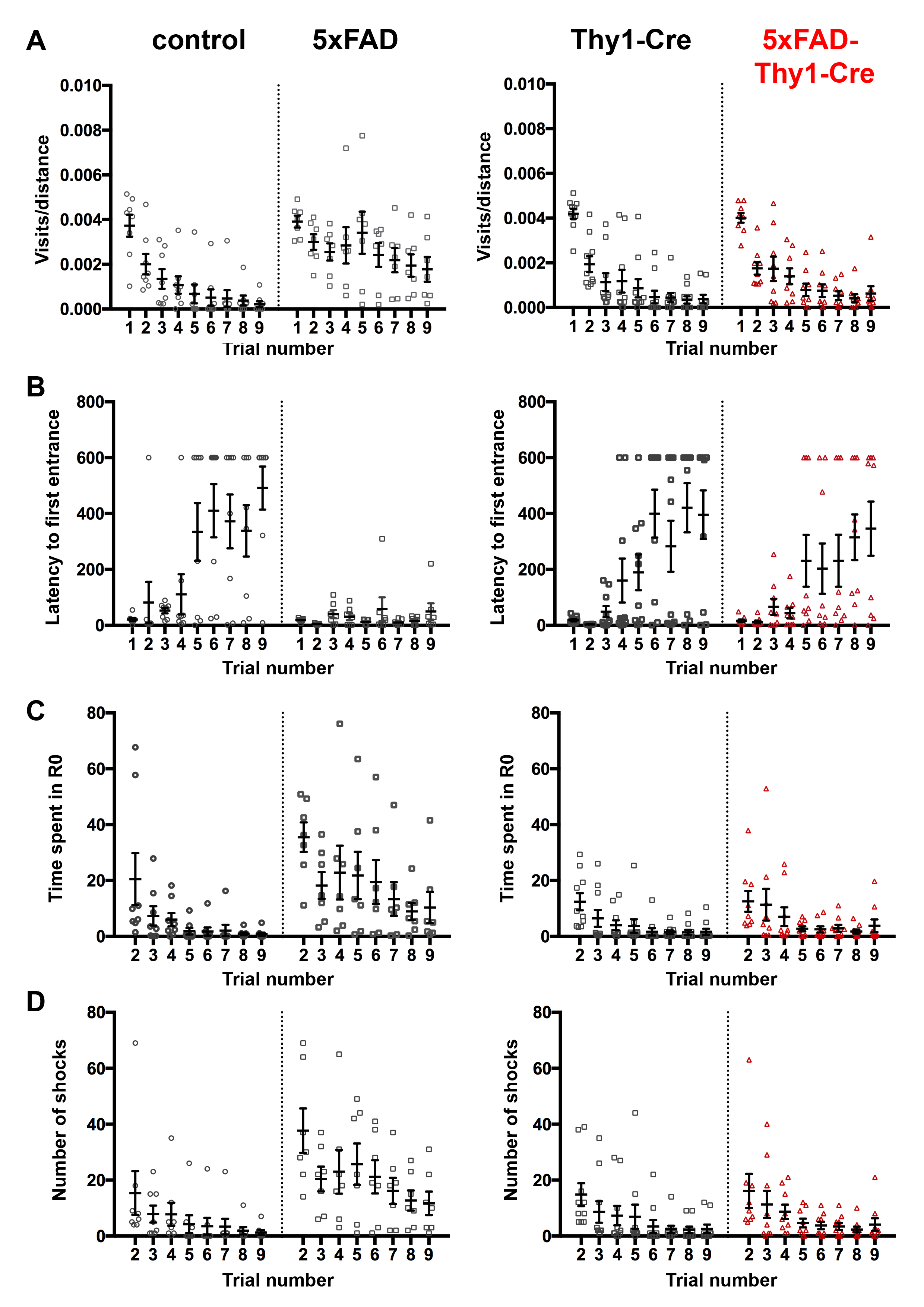

Supplement: Supplementary file 6 [file Image_5.TIF]

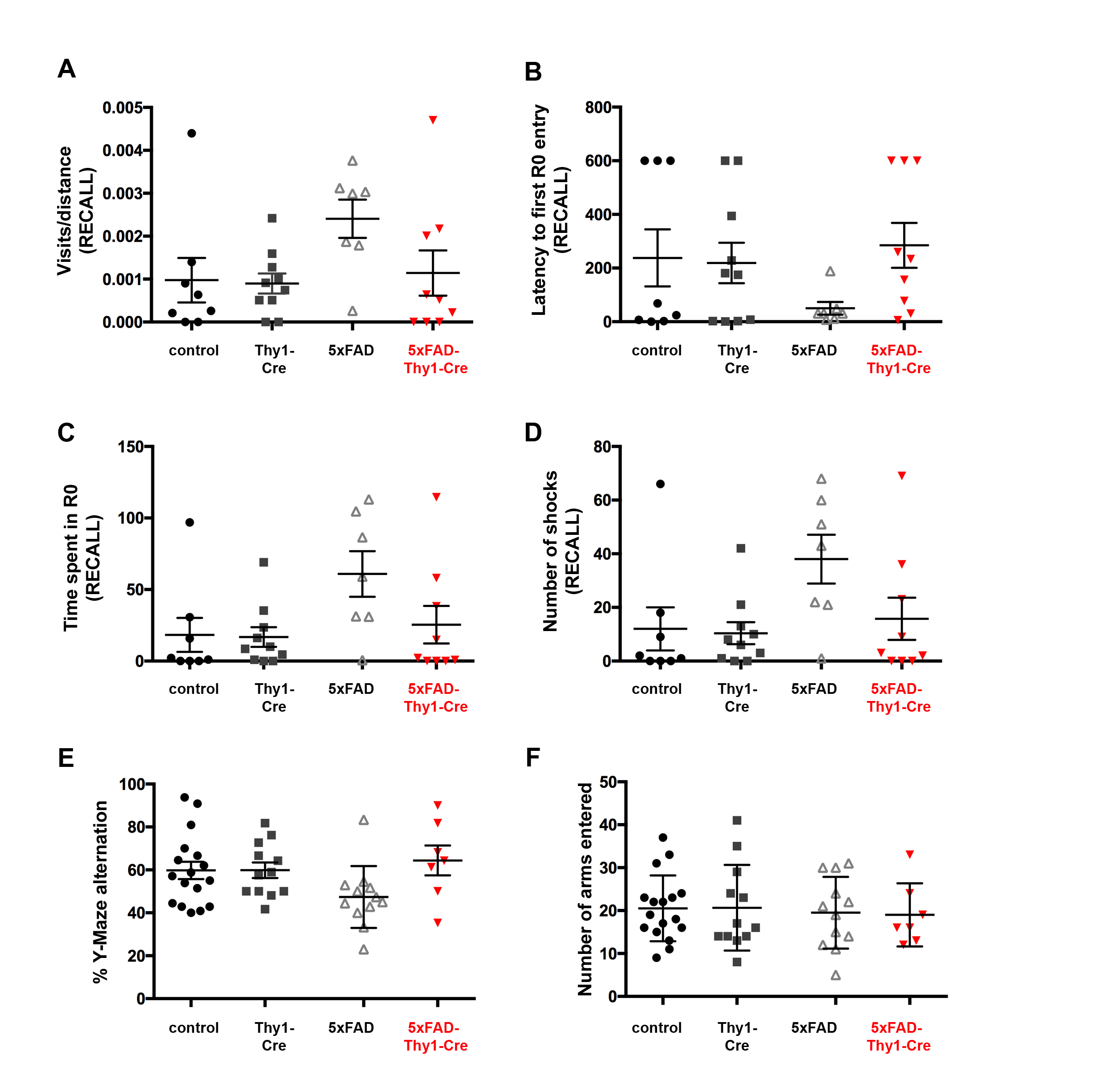

Supplement: Supplementary file 7 [file Image_6.TIF]

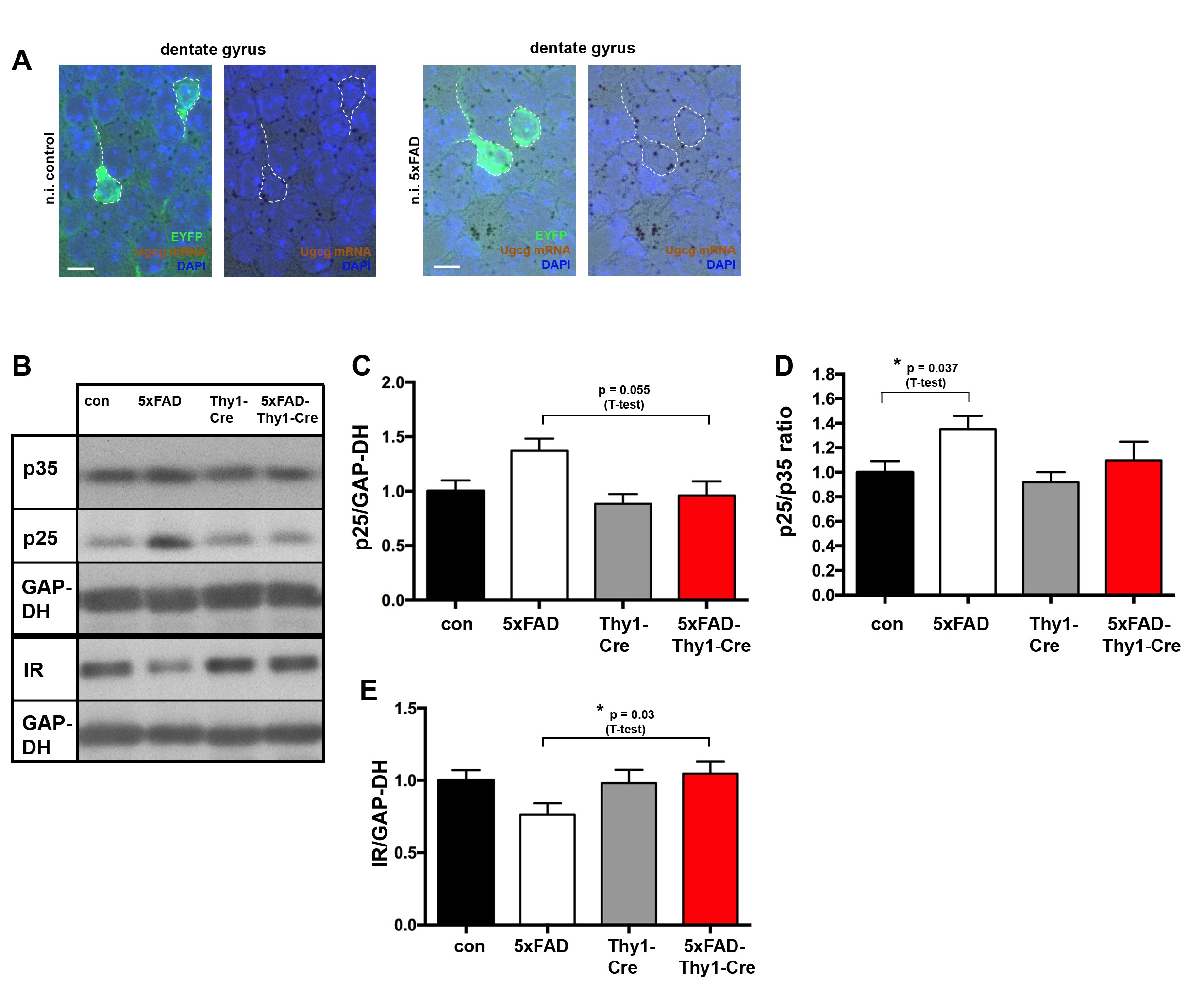

Supplement: Supplementary file 8 [file Image_7.TIF]

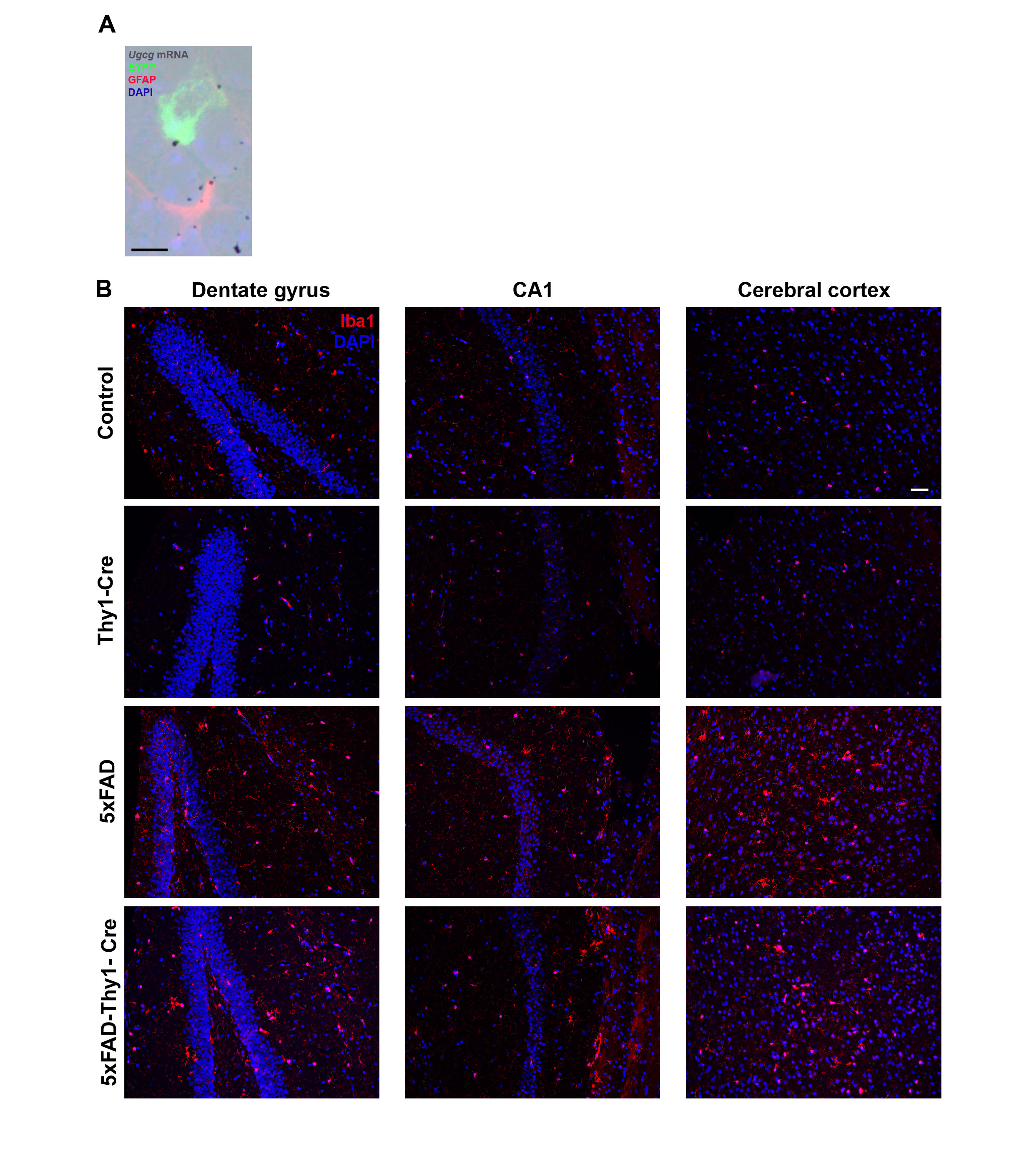

Supplement: Supplementary file 9 [file Image_8.TIF]
